# A Data-Driven Framework for Identifying Intensive Care Unit Admissions Colonized With Multidrug-Resistant Organisms

**DOI:** 10.3389/fpubh.2022.853757

**Published:** 2022-03-17

**Authors:** Çaǧlar Çaǧlayan, Sean L. Barnes, Lisa L. Pineles, Anthony D. Harris, Eili Y. Klein

**Affiliations:** ^1^Asymmetric Operations Sector, Johns Hopkins University Applied Physics Laboratory, Laurel, MD, United States; ^2^Department of Decision, Operations and Information Technologies (DO&IT), R.H. Smith School of Business, University of Maryland, College Park, MD, United States; ^3^Department of Epidemiology and Public Health, University of Maryland School of Medicine, Baltimore, MD, United States; ^4^Department of Emergency Medicine, Johns Hopkins University School of Medicine, Baltimore, MD, United States; ^5^Center for Disease Dynamics, Economics and Policy, Washington, DC, United States

**Keywords:** multidrug-resistant organisms (MDROs), carbapenem-resistant *Enterobacteriaceae* (CRE), vancomycin-resistant enterococci (VRE), Methicillin-resistant *Staphylococcus aureus* (MRSA), healthcare-associated infections (HAIs), machine learning (ML), data-centric analytics, predictive analytics

## Abstract

**Background:**

The rising prevalence of multi-drug resistant organisms (MDROs), such as Methicillin-resistant *Staphylococcus aureus* (MRSA), Vancomycin-resistant *Enterococci* (VRE), and Carbapenem-resistant *Enterobacteriaceae* (CRE), is an increasing concern in healthcare settings.

**Materials and Methods:**

Leveraging data from electronic healthcare records and a unique MDRO universal screening program, we developed a data-driven modeling framework to predict MRSA, VRE, and CRE colonization upon intensive care unit (ICU) admission, and identified the associated socio-demographic and clinical factors using logistic regression (LR), random forest (RF), and XGBoost algorithms. We performed threshold optimization for converting predicted probabilities into binary predictions and identified the cut-off maximizing the sum of sensitivity and specificity.

**Results:**

Four thousand six hundred seventy ICU admissions (3,958 patients) were examined. MDRO colonization rate was 17.59% (13.03% VRE, 1.45% CRE, and 7.47% MRSA). Our study achieved the following sensitivity and specificity values with the best performing models, respectively: 80% and 66% for VRE with LR, 73% and 77% for CRE with XGBoost, 76% and 59% for MRSA with RF, and 82% and 83% for MDRO (i.e., VRE or CRE or MRSA) with RF. Further, we identified several predictors of MDRO colonization, including long-term care facility stay, current diagnosis of skin/subcutaneous tissue or infectious/parasitic disease, and recent isolation precaution procedures before ICU admission.

**Conclusion:**

Our data-driven modeling framework can be used as a clinical decision support tool for timely predictions, characterization and identification of high-risk patients, and selective and timely use of infection control measures in ICUs.

## Introduction

The increasing prevalence of multidrug resistant organisms (MDROs), bacteria that are resistant to one or more classes of antibiotics, is an increasingly concerning issue in the community, and in particular, in healthcare settings, where admitted patients are especially susceptible to developing an infection (1–3). These organisms (also known as multidrug-resistant bacteria) pose a significant threat to patient safety in the form of healthcare-associated (i.e., nosocomial) infections (HAIs) (4), which are associated with considerable morbidity, mortality, and healthcare costs (5), and have the potential to spread within the community (6, 7).

Two MDROs that are the most prevalent causes of HAIs are Methicillin-resistant *Staphylococcus aureus* (MRSA) and vancomycin-resistant *Enterococcus* (VRE) (8, 9), which are currently classified as serious threats by the U.S. Centers for Disease Control and Prevention (CDC) (10). MRSA is reported to cause an estimate of 80,461 infections and 11,285 deaths per year, and VRE is estimated to cause 20,000 infections and 11,300 deaths per year (1), with both MDROs being associated with poor treatment outcomes following infections (11, 12), longer length of hospitalization, and higher healthcare costs (13–15).

In recent years, Carbapenem-resistant *Enterobacteriaceae* (CRE), an MDRO class that is highly resistant to carbapenems and other antibiotics reserved for treatment of severe infections, have reached concerning levels in healthcare facilities in the U.S. (16), and around the world (17). This trend has prompted the CDC to classify CRE as an urgent threat to public health, its highest risk category (1). CRE is currently less prevalent than MRSA and VRE, estimated to cause 9,000 infections and 600 deaths per year (1), but is an immediate public health threat because infections caused by CRE (e.g., pneumonia, urinary tract infections, bloodstream infections and wound infections) are very difficult to treat (18, 19) and have been associated with poor treatment outcomes (20–23), and high costs (24).

Besides the high morbidity and mortality rates, multidrug-resistant pathogens can also place a heavy economic burden on individual healthcare facilities, as well as on the entire U.S. healthcare system. Among other factors, MDRO-related costs are increased due to prolonged hospital stay, additional treatments, post-discharge complications, and implemented infection control measures including the set-up of isolation wards and cleaning or replacement of contaminated materials (25). In particular, earlier studies reported average additional hospital costs attributable to each VRE infection as high as $77,558, whereas the lower bound estimate was around $10,000 (in 2003) (14, 26). Despite its lower prevalence, a single CRE infection was also estimated to be costly for hospitals ($22,484–$66,031), and third-party payers ($10,440–$31,621). Further, including out-of-pocket costs and labor and productivity losses, CRE was estimated to cost society $37,778–$83,512 per infection (24). Finally, averaging around $60,000–$70,000 per infected patient, total healthcare spending for MRSA was estimated to be around $10 billion per year in the U.S. (27). These estimates not only show the heavy financial burden of MDROs at an individual and a population level, but also demonstrate the value of prevention, early detection, and early intervention. If MDRO colonization are detected and intervened upon before they harm patients and drive up costs, then the valuable resources spent for MDRO treatments (28) could be allocated to other pressing public health problems for the greater good of the U.S. society.

Colonized patients carry an MDRO at a detectable level, meaning that a cultured swab sample would test positive, but the patient would not show clinical indications (i.e., signs or symptoms) of illness caused by an MDRO. Harboring MDROs, these patients are at a risk for subsequent infection, as a significant fraction of MDRO colonization will eventually cause clinically apparent infections that are difficult and costly to treat (28–30). They also pose a threat to other patients, as healthcare workers who interact with these patients can become contaminated with the organism and transmit it to other patients. As a result, it is important to rapidly identify and then monitor colonized patients to reduce the risk of disease transmission and subsequent infections (31).

The importation of MDROs into hospitals and other healthcare settings is a major determinant for (the rate and magnitude of) transmission and outbreak (32–34). Among hospital departments, intensive care units (ICUs) are the wards where the prevalence of MDROs has reported to be higher (35, 36). Further, patients admitted to the ICUs are more vulnerable to develop infections from these organisms (37, 38). Accordingly, ICUs have become a central point of focus for the control and prevention of MDRO colonization and infection within hospitals (39).

A variety of interventions have been proposed and implemented in order to prevent the transmission of MDROs in ICUs. Effective and commonly utilized interventions include (i) hand hygiene, especially when healthcare workers contact colonized or infected patients (40), (ii) contact precautions (e.g., wearing gloves and gowns) when caring for colonized or infected patients (41), and (iii) isolation or cohorting of colonized or infected patients (42). Despite their effectiveness, however, these preventive measures are often not applied in a timely manner due to imperfect compliance and the delay (or even failure) to detect patients colonized with an MDRO (9).

Surveillance for MDRO colonization is an instrumental practice for detecting patients who may require an intervention (43, 44). Yet, the implementation and cost-effectiveness of universal (i.e., active) surveillance and testing strategies, such as screening of all newly admitted ICU patients, has been a controversial topic (45). Some critics argue that the costs associated with universal screening, including the opportunity costs of the human and physical resources being utilized, are likely to outweigh the benefits of active surveillance (46). Accordingly, universal surveillance of all patients may not be feasible to implement in many healthcare facilities due to resource constraints (47–49). Instead, targeted surveillance strategies, which offer a cost-effective compromise for detecting asymptomatic colonization, have been advocated by national guidelines (50–52) when a sufficiently accurate method for identifying high-risk individuals is available. Accordingly, rapid and accurate identification of patients who are at high risk for MDRO colonization is critical for timely and targeted implementation of screening protocols and other preventive measures, as well as administration of appropriate treatments (e.g., avoiding the misuse of antibiotics).

Given the aforementioned challenges, a system that facilitates timely and reliable identification of newly admitted patients who are likely to be colonized with an MDRO would be quite useful to improve patient safety and effective utilization of critical hospital resources (53). By accurately identifying significant risk factors, this system can help define high-risk subpopulations and hence, could enable the implementation of a cost-effective targeted screening program. Moreover, if highly predictive, it can further be used to immediately initiate clinical interventions, such as contact precautions, as soon as a high-risk individual is admitted to the ICU. Such a real-time system would be particularly useful in ICUs because, currently, identification of colonized patients relies on costly and labor intensive clinical laboratory results that usually require at least 1–2 days to process and hence, delay subsequent necessary actions to prevent and control the spread of MDROs.

A particular challenge for the design of a reliable prediction framework is the class imbalance problem that is commonly observed in clinical datasets. Clinical datasets are often not balanced in their class labels, where the predictors and/or prediction outcomes do not make up an equal portion of the data. The imbalance can be particularly large when the prediction outcomes are MDROs, as their prevalence is usually <15% and can be as low as <2% as observed in our data. Given that ignoring the class imbalance, especially when it is large, yields poor predictions, it is necessary to consider and address this challenge up front while developing a prediction framework for accurate and reliable results.

In this study, we developed a data-driven framework to identify patients who are likely to be colonized with VRE, CRE, or MRSA upon ICU admission, leveraging 2 years of electronic health record (EHR) data from a large academic medical center. The objective of our study was to develop a modeling framework that can cope with significant class imbalance, commonly observed in clinical datasets, and can be used (1) to generate timely and accurate predictions for newly admitted ICU patients, and (2) to identify the key socio-demographic and clinical factors affecting the incidence of MDRO colonization. The developed framework relied on three supervised machine learning algorithms (namely, regularized logistic regression, random forest, and XGBoost), which were trained on the EHR data to make timely and accurate predictions for the patients newly admitted to the ICU.

Our study achieved the following results for the primary MDRO colonization outcomes: 80% sensitivity and 66% specificity for VRE, 73% and 77% for CRE, 76% and 59% for MRSA, and 82% and 83% for colonization with any MDRO (i.e., VRE, CRE, or MRSA). Moreover, our modeling approach identified long-term care facility stay, current diagnosis of skin/subcutaneous tissue conditions or infectious/parasitic disease, and recent isolation precaution procedures before ICU admission as key predictors. The proposed modeling framework was able to detect over 80% of positive MDRO cases upon ICU admission with less than a 20% false-positive rate, which would enable timely and targeted implementation of preventive measures for infection control in ICUs.

Currently most hospitals lack (or choose not implement) universal screening programs for MDROs. The practical utility and impact of this study was to translate EHR data into insights and real-time predictions to effectively guide VRE, CRE, and MRSA-related infection control decisions in ICUs. The means to achieve this impact was to build a robust predictive analytics framework that produces reliable and evidence-based predictions with high sensitivity, ensuring timely detection of MDRO colonization, and high specificity, preventing inefficient use of limited resources. This was the primary objective of our study. Once thoroughly and externally validated, this modeling framework would allow hospitals to implement a clinical decision support system that could serve as a cost-effective universal MDRO screening tool at ICU admission without using any hospital resources except for EHR data.

The remainder of this article is organized as follows: In Section Materials and Methods, we present our data and describe our methodology. In particular, in Section Data Description, we introduce our data and describe the clinical and socio-demographic predictors included in our models. Then, in Section Prediction Models, Model Training and Validation, and Threshold Optimization, we introduce the predictive models and describe the techniques we utilize to improve prediction accuracy and address class imbalance. In Section Results, we present our prediction results and report the key predictors for MDRO colonization in our data set. In Section Discussions, we summarize our results, and discuss the policy implications of our approach and findings. Finally, in Section Conclusion and Future Work, we propose directions for future research, and conclude our study.

## Materials and Methods

In this section, we first describe our data source, in Section Data Description, and present the variables and prediction outcomes in our dataset. Then, in Section Prediction Models, Model Training and Validation, and Threshold Optimization, we introduce our modeling framework and describe our methods. In particular, first, we introduce the prediction models we used, and then, discuss our model specification (training) and performance evaluation (testing) stages, describing how we performed hyperparameter tuning, stratified cross-validation, threshold optimization, and finally, out-of-sample evaluations.

### Data Description

In this study, we used electronic healthcare record (EHR) data from the University of Maryland Medical Center (UMMC), an academic teaching hospital located in Baltimore, Maryland. Our dataset contained records for 3,958 patients admitted to a surgical or medical ICU in 2017 or 2018. In total, we observed 4,670 individual admissions. Our dataset included the following variables: (1) hospital admission source and type, (2) age, (3) sex, (4) race and ethnicity, (5) region/state of residency, (6) total time of prior ICU stays and hospital inpatient stays within the previous year, (7) prior antibiotic prescriptions, (8) diagnoses for prior hospital and/or ICU stays within the previous year, (9) diagnoses for current hospital stay before ICU admission, (10) surgical and medical procedures conducted during prior hospital and/or ICU stays within the previous year, and (11) recent procedures conducted for current hospital stay prior to ICU admission. We treated all predictors utilized in the models as categorical. Descriptive statistics regarding these variables and their categories can be found in the Supplementary Material (Appendix A).

The prediction outcomes were colonization with VRE, CRE, or MRSA upon ICU admission, both separately and as an aggregate (union) outcome. Conducting active surveillance in the ICUs, UMMC screened newly admitted patients for colonization upon admission and periodically during their stay. At UMMC, active surveillance involves taking routine peri-rectal cultures for VRE and nasal cultures for MRSA on all patients admitted to an ICU at the time of admission, weekly, and upon discharge. CRE detection was also primarily done *via* perirectal swabs and also included clinical cultures (e.g., blood, urine, wound cultures). We identified the positive (i.e., colonized) and negative (i.e., uncolonized) results based on the laboratory tests conducted within 2 days (i.e., both before and after) of ICU admissions. We limited the time window for the screening results within 2 days (54, 55) in an attempt to avoid inclusion of acquisition cases, for which initially susceptible (i.e., colonization-free) patients acquire an MDRO during their ICU stay. Screening outcomes were not available for all 4,670 ICU admissions. The total number of screening results available was 3,860 for VRE, 3,661 for CRE, 4,446 for MRSA, and 4,503 for MDRO. In the dataset, 503 (13.03%) of ICU admissions tested positive for VRE, 53 (1.45%) for CRE, 332 (7.47%) for MRSA, and 792 (17.59%) for any one of these MDROs.

In the UMMC dataset, all prior and current diagnoses were coded using the International Statistical Classification of Diseases and Related Health Problems (ICD)-10 codification. We used the Agency for Healthcare Research and Quality's Clinical Classifications Software (CCS) to further categorize the prior and current diagnoses that were present on admission (PoA). The CCS is a diagnosis and procedure categorization catalog (https://www.hcup-us.ahrq.gov/toolssoftware/ccs10/ccs10.jsp>), mapping the ICD-10 diagnosis codes into 18 categories: (1) Infectious and parasitic diseases, (2) Neoplasms, (3) Endocrine, nutritional, and metabolic diseases and immunity disorders, (4) Diseases of the blood and blood-forming organs, (5) Mental illness, (6) Diseases of the nervous system and sense organs, (7) Diseases of the circulatory system, (8) Diseases of the respiratory system, (9) Diseases of the digestive system, (10) Diseases of the genitourinary system, (11) Complications of pregnancy, childbirth, and the puerperium, (12) Diseases of the skin and subcutaneous tissue, (13) Diseases of the musculoskeletal system and connective tissue, (14) Congenital anomalies, (15) Certain conditions originating in the perinatal period, (16) Injury and poisoning, (17) Symptoms, signs, and ill-defined conditions and factors influencing health status, and (18) Residual or unclassified codes.

We labeled a procedure as recent if it was performed during the current hospital stay. We recorded all recent procedures performed in the hospital inpatient settings prior to the current ICU admission with respect to the ICD-10 Procedure Coding System (PCS), for which each character has a categorical indication. Using the first character of the ICD-10 PCS codes, we classify the recent procedures into eight categories as follows: (i) Medical and Surgical (“0”), (ii) Placement (“2”), (iii) Administration (“3”), (iv) Measurement and Monitoring (“4”), (v) Extracorporeal or Systemic Procedures (“5” and “6”), (vi) Other Procedures (“8”), (vii) Imaging (“B”), and (viii) Other/Miscellaneous (“1”, “7”, “9”, “C”, “D”, “F”, “G”, and “X”). Further, using the first two characters of the ICD-10 PCS codes, we also map the recent procedures into 44 categories (see Supplementary Material Appendix A). In our analysis, we include both the single- and double-character based categorizations so that our algorithms can learn which specifications are more important for predicting our MDRO outcomes. We classified prior hospital procedures having the ICD-10 PCS codes in a similar manner as the recent procedures.

Prior outpatient procedures were recorded using the Current Procedural Terminology (CPT) system (*https://www.ama-assn.org/amaone/cpt-current-procedural-terminology*), which we classified into 6 categories: (i) Evaluation and Management, (ii) Anesthesia (iii) Medicine (iv) Radiology (v) Pathology and Laboratory, and (vi) Surgery. The CPT codes for surgery include 18 sub-types, enabling us to construct a more detailed categorization with 23 classes. We used both the 6-class and 23-class CPT codes as predictors for our descriptive and predictive analyses.

### Prediction Models, Model Training and Validation, and Threshold Optimization

A variety of techniques have been utilized to analyze complex disease dynamics and quantify its parameters (e.g., the estimation of transmission rate), identify risk factors, and assess the impact of infection control strategies (56). These approaches include prediction modeling, computational simulation, and analytic-formula based models such as decision trees (57), artificial neural network (58), agent-based simulation for a hospital ward (59, 60) or healthcare system (61), dynamic patient and healthcare worker networks (62–64), compartmental systems dynamics models (based on ordinary differential equations) (65, 66), (approximate) Bayesian (computation) techniques (67), and Markov chain based approaches (68, 69). Among these techniques, data-driven prediction models, such as the ones we used in this study, are particularly valuable tools for generating real-time predictions, identifying the significant risk factors, and quantifying their impact on the outcomes of interest (70). In addition to these modeling-based approaches, there is also rich clinical literature studying MDRO colonization. See the Supplementary Material (Appendix B) for a summary of the clinical studies that assessed the risk factors associated with MDRO colonization, and developed simple clinical prediction rules based on the identified predictors.

We utilized three supervised machine learning (ML) algorithms to predict colonized patients upon ICU admission and to identify significant clinical and socio-demographic factors associated with the outcomes of interest: (1) logistic regression (LR) (71, 72), (2) random forest (RF) (73), and (3) extreme gradient boosting (XGBoost) (74). To perform regularization and feature selection for our logistic regression models, we used least absolute shrinkage and selection operator (LASSO), which was originally developed for linear regression (75) and then applied to other algorithms including LR (76).

**Figure 1:**
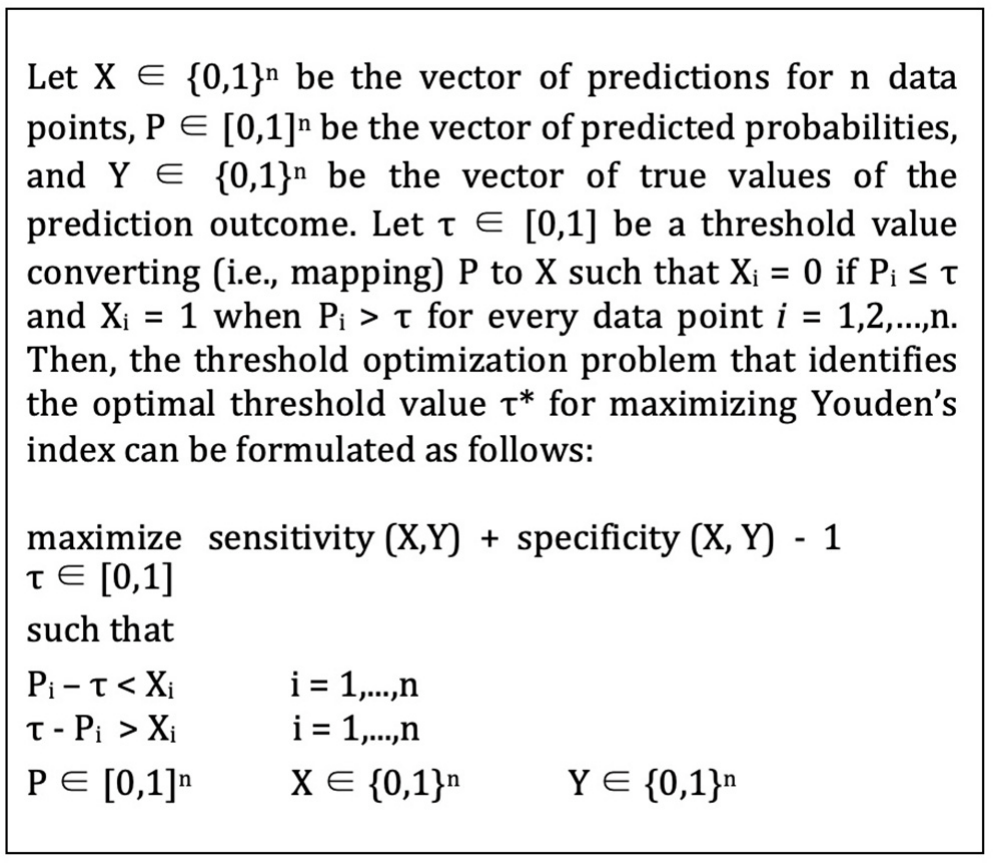
Threshold optimization formulation.

For each model, we split the data into an 80% subset for model training and cross-validation and a 20% subset for out-of-sample evaluation. We used a 10-fold stratified cross-validation scheme both for hyperparameters tuning for the algorithms and threshold optimization for the conversion of predicted colonization risks into binary predictions (see Figure 1). We selected the 10-fold due to the relatively small sample size of our data, in an effort to preserve as much data as possible for model development and training. We selected the stratified scheme to account for the class imbalance in our data, which preserves a proportion of the positive outcome for each fold similar to the complete dataset.

We defined a grid search for a core set of hyperparameters for each algorithm, and used the area under the receiver operating characteristic curve (AUC) as the objective function to maximize (out-of-sample) model performance. We selected the hyperparameters achieving the highest mean AUC across the 10 folds for model training. In particular, the hyperparameters were optimized and fine-tuned by the function “*LogisticRegressionCV*” for LR, and “*GridSearchCV*” for RF and XGBoost. For each machine learning algorithm, we summarize the hyperparameters and model parameters corresponding to the best performing machine learning models of our study in Tables 1–3. The programming code samples of the supervised ML algorithms utilized in this study are also provided in the Supplementary Material (Appendix D).

**Table 1:** Model parameters for best performing logistic regression models.

**Best models-logistic regression**				
**Parameter**	**VRE**	**CRE**	**MRSA**	**MDRO**
Cs	100	100	100	100
class_weight	None	None	None	None
cv=StratifiedKFold	n_splits=10	n_splits=10	n_splits=10	n_splits=10
dual	False	False	False	False
fit_intercept	True	True	True	True
intercept_scaling	1	1	1	1
max_iter	100	100	100	100
multi_class	'ovr'	'ovr'	'ovr'	'ovr'
n_jobs	1	1	1	1
penalty	L1	L1	L1	L1
random_state	None	None	None	None
refit	True	True	True	True
scoring	roc_auc	roc_auc	roc_auc	roc_auc
solver	liblinear	liblinear	liblinear	liblinear
tol	0.0001	0.0001	0.0001	0.0001
verbose	0	0	0	0
Threshold Bound	0.15	0.025	0.20	0.50

**Table 2:** Model parameters for best performing random forest models.

**Best models-random forest**				
**Parameter**	**VRE**	**CRE**	**MRSA**	**MDRO**
cv=StratifiedKFold	n_splits=10	n_splits=10	n_splits=10	n_splits=10
estimator=RandomForestClassifier	Yes	Yes	Yes	Yes
bootstrap	True	True	True	True
max_depth	None	None	None	None
max_leaf_nodes	None	None	None	None
min_impurity_decrease	0	0	0	0
init_min_samples_leaf	1	1	1	1
init_min_samples_split	2	2	2	2
n_estimators	200	200	200	200
n_jobs	4	4	4	4
param_grid={'min_samples_leaf'}	[5, 10,..., 250]	[5, 10,..., 250]	[5, 10,..., 250]	[5, 10,..., 250]
param_grid={pre_dispatch}	2*n_jobs	2*n_jobs	2*n_jobs	2*n_jobs
param_grid={scoring}	roc_auc	roc_auc	roc_auc	roc_auc
optimal_min_samples_leaf	5	30	10	5
Threshold Bound	0.20	0.05	0.30	0.40

**Table 3:** Model parameters for best performing XGBoost models.

**Best models-XGBoost**				
**Parameter**	**VRE**	**CRE**	**MRSA**	**MDRO**
colsample_bytree	0.8	0.8	0.8	0.8
gamma	0	0	0	0
learning_rate	0.05	0.05	0.05	0.05
max_depth	5	5	5	5
min_child_weight	1	1	1	1
n_estimators	200	200	200	200
nthread	4	4	4	4
objective	binary:logistic	binary:logistic	binary:logistic	binary:logistic
seed	1337	1337	1337	1337
subsample	0.8	0.8	0.8	0.8
Threshold Bound	0.15	0.015	0.10	0.30

**Figure 2:**
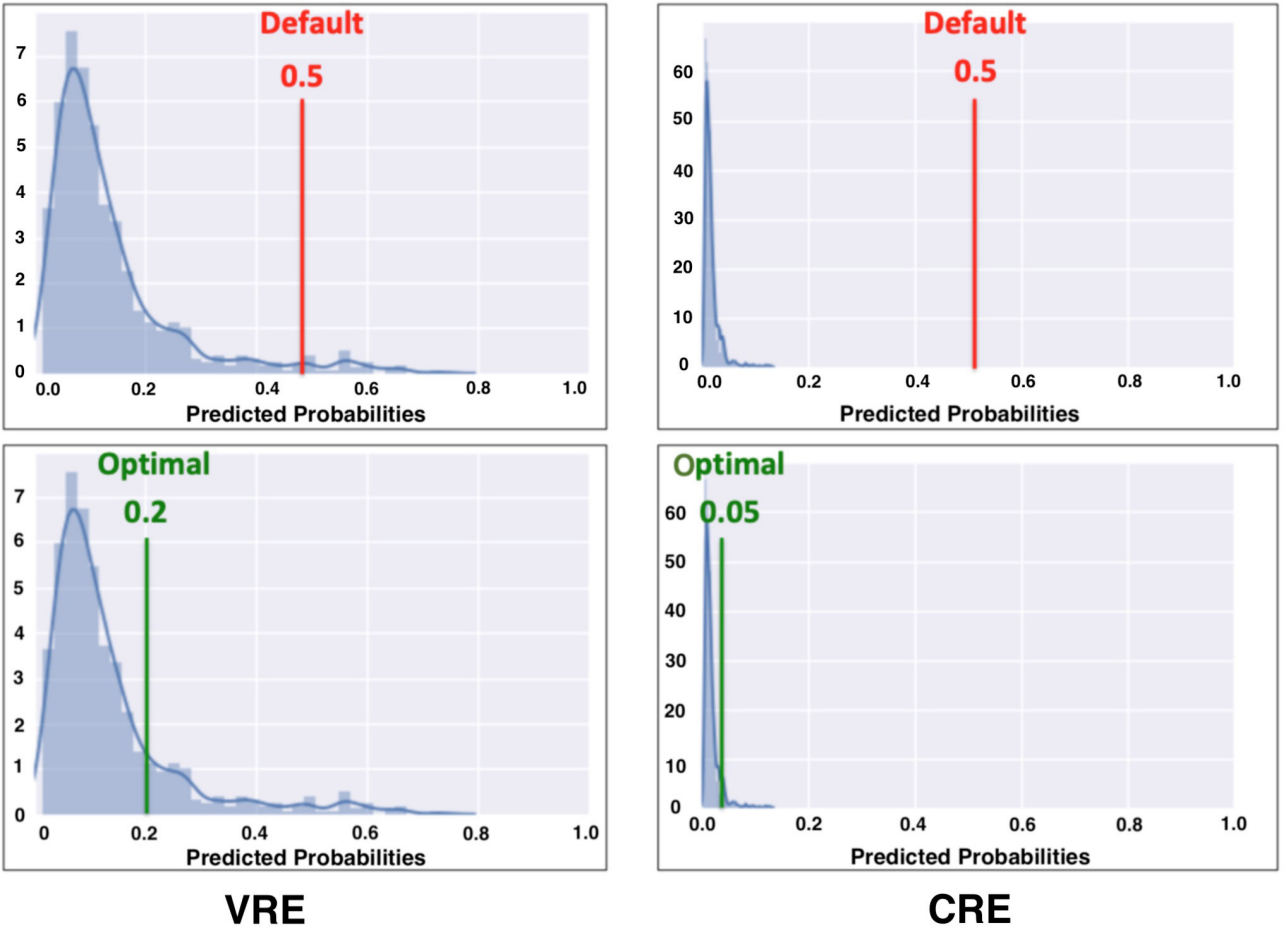
Threshold value for converting predicted probabilities to binary predictions.

After choosing the hyperparameters, the next step of the model specification was to identify the ideal cut-off (i.e., optimal threshold) value for converting predicting probabilities into binary predictions. As an initial output, the ML algorithms generate predicted probabilities for the training instances, indicating how likely each patient to be colonized with an MDRO. These predicted probabilities are then translated into binary prediction outcomes using a threshold value. Specifically, observations for which the predicted probabilities are greater than this threshold, denoted as τ, are classified as positive (i.e., colonized), and otherwise, the patient is assigned to the negative (i.e., susceptible) class. Given the class imbalance observed in our dataset, the default threshold value of 0.5 was unlikely to be effective for our study (see Figure 2). Consequently, we performed an optimization (77) to search for the best threshold that classifies the predicted probabilities while maximizing the Youden Index (i.e., sensitivity + specificity - 1) for out-of-sample predictions (78).

We performed the threshold optimization using the same 10-fold stratified cross validation scheme used for the hyperparameter tuning. The optimal threshold was determined for each fold using the in-sample predicted probabilities from the 90% subset of training data. Then, we evaluated the performance (i.e., Youden's index) of this threshold over the 10% subset. We repeated this process for each fold, and selected the mean of these 10 optimal thresholds as the final cut-off value. We used a bounded numerical search algorithm to solve the optimization problem (79), using a lower bound of zero and varying the upper bound for each algorithm to ensure an effective threshold is found. It is noteworthy to emphasize that the upper bound values we considered for each specific outcome were different because the prevalence of the colonized (i.e., positive) instances among VRE, CRE, MRSA, and MDRO were different, which directly affected the outcome of the threshold optimization procedure.

Model specification was completed when we determined the hyperparameters, chose the threshold value (for each model), and re-trained the models on the full (80%) training set. Next, we evaluated the (out-of-sample) performance of the trained models on the (20%) test sets, reporting the AUC, sensitivity, and specificity values obtained. For each MDRO, we conducted a systematic numerical experiment with a range of upper bound values for threshold optimization, and obtained predictions with varying sensitivity and specificity values for VRE, CRE, MRSA, and MDRO (the aggregate prediction outcome). We provide these results in Section Discussions for each outcome (e.g., VRE) and algorithm (e.g., XGBoost), and separately, discuss the best performing models for each MDRO.

We also used our modeling framework to identify the key socio-demographic and clinical factors for predicting colonization with VRE, CRE, and MRSA separately and in aggregate. For the LR models, we used odds ratios (ORs), which quantify the associated increase (for values >1) or decrease (for values <1) in the likelihood of colonization. For the tree-based models (i.e., RF and XGBoost), we used feature importance (FI), which quantifies the relative frequency that each factor is used to construct the ensemble. Using these two metrics (i.e., OR and FI), we ordered the identified predictors for each MDRO and report the top five key predictors that are highly ranked across all of the best performing ML models, calculated by the average ranking across the best models.

## Results

In a total of 4,670 ICU admissions corresponding to 3,958 patients examined, the rate of colonization was 17.59% for MDRO (13.03% VRE, 1.45% CRE, and 7.47% MRSA). This study separately predicted VRE, CRE, and MRSA colonization upon ICU admission. In addition, combining these three antibiotic-resistant bacteria, the models we developed also predicted colonization with any of these MDROs (i.e., VRE, CRE, or MRSA) upon ICU admission without specifying the particular organism. As a result, our modeling framework generated separate predictions for four cases (namely, VRE, CRE, MRSA, and MDRO) using logistic regression (with LASSO regularization), random forest, and XGBoost algorithms. In Table 4, we summarize the model results for these four outcomes under different upper bound values corresponding to the threshold optimization process.

**Table 4:** Performance summary of the machine learning models for VRE, CRE, MRSA, and MDRO colonization predictions.

**Threshold opt. upper bound = 0.05**	**VRE (503/3860** **=** **13.03%)**			**Threshold opt. upper bound = 0.010**	**CRE (53/3661** **=** **1.45%)**			**Threshold opt. upper bound = 0.03**	**MRSA (332/4446** **=** **7.47%)**			**Threshold opt. upper bound = 0.1**	**MDRO (792/4503** **=** **17.59%)**			
	**Log**.	**XGBoost**	**Rand**.		**Log**.	**XGBoost**	**Rand**.		**Log**.	**XGBoost**	**Rand**.		**Log**.	**XGBoost**	**Rand**.	**Dec**.
	**Reg**.		**Forest**		**Reg**.		**Forest**		**Reg**.		**Forest**		**Reg**.		**Forest**	**Tree**
Training AUC	0.76	0.77	0.77	Training AUC	0.70	0.76	0.78	Training AUC	0.65	0.66	0.66	Training AUC	0.72	0.86	0.88	0.75
Testing AUC	0.80	0.77	0.77	Testing AUC	0.78	0.72	0.71	Testing AUC	0.66	0.66	0.69	Testing AUC	0.70	0.87	0.89	0.76
Testing sensitivity	0.99	0.97	1.00	Testing sensitivity	1.00	0.73	0.82	Testing sensitivity	1.00	0.88	1.00	Testing sensitivity	0.92	0.97	1.00	0.93
Testing specificity	0.09	0.33	0.15	Testing specificity	0.31	0.68	0.37	Testing specificity	0.02	0.22	0.00	Testing specificity	0.30	0.39	0.21	0.51
**Threshold opt. upper bound** **=** **0.1**	**VRE (503/3860** **=** **13.03%)**			**Threshold opt. upper bound** **=** **0.015**	**CRE (53/3661** **=** **1.45%)**			**Threshold opt. upper bound** **=** **0.075**	**MRSA (332/4446** **=** **7.47%)**			**Threshold opt. upper bound** **=** **0.2**	**MDRO (792/4503** **=** **17.59%)**			
	**Log**.	**XGBoost**	**Rand**.		**Log**.	**XGBoost**	**Rand**.		**Log**.	**XGBoost**	**Rand**.		**Log**.	**XGBoost**	**Rand**.	**Dec**.
	**Reg**.		**Forest**		**Reg**.		**Forest**		**Reg**.		**Forest**		**Reg**.		**Forest**	**Tree**
Training AUC	0.76	0.77	0.76	Training AUC	0.70	0.76	0.80	Training AUC	0.65	0.66	0.67	Training AUC	0.72	0.86	0.88	0.76
Testing AUC	0.80	0.77	0.77	Testing AUC	0.78	0.72	0.71	Testing AUC	0.66	0.66	0.71	Testing AUC	0.70	0.87	0.89	0.81
Testing sensitivity	0.89	0.79	0.88	Testing sensitivity	0.82	0.73	0.73	Testing sensitivity	0.76	0.71	0.82	Testing sensitivity	0.68	0.82	0.90	0.93
Testing specificity	0.49	0.57	0.48	Testing specificity	0.57	0.77	0.51	Testing specificity	0.45	0.53	0.45	Testing specificity	0.59	0.74	0.69	0.58
**Threshold opt. upper bound** **=** **0.15**	**VRE (503/3860** **=** **13.03%)**			**Threshold opt. upper bound** **=** **0.020**	**CRE (53/3661** **=** **1.45%)**			**Threshold opt. upper bound** **=** **0.1**	**MRSA (332/4446** **=** **7.47%)**			**Threshold opt. upper bound** **=** **0.3**	**MDRO (792/4503** **=** **17.59%)**			
	**Log**.	**XGBoost**	**Rand**.		**Log**.	**XGBoost**	**Rand**.		**Log**.	**XGBoost**	**Rand**.		**Log**.	**XGBoost**	**Rand**.	**Dec**.
	**Reg**.		**Forest**		**Reg**.		**Forest**		**Reg**.		**Forest**		**Reg**.		**Forest**	**Tree**
Training AUC	0.76	0.77	0.76	Training AUC	0.70	0.76	0.80	Training AUC	0.65	0.66	0.66	Training AUC	0.72	0.86	0.87	0.76
Testing AUC	0.80	0.77	0.78	Testing AUC	0.78	0.72	0.73	Testing AUC	0.66	0.66	0.68	Testing AUC	0.70	0.87	0.89	0.81
Testing sensitivity	0.80	0.73	0.78	Testing sensitivity	0.73	0.55	0.73	Testing sensitivity	0.64	0.67	0.73	Testing sensitivity	0.65	0.75	0.85	0.89
Testing specificity	0.66	0.65	0.59	Testing specificity	0.69	0.83	0.63	Testing specificity	0.60	0.60	0.57	Testing specificity	0.63	0.82	0.79	0.65
**Threshold opt. upper bound** **=** **0.2**	**VRE (503/3860** **=** **13.03%)**			**Threshold opt. upper bound** **=** **0.025**	**CRE (53/3661** **=** **1.45%)**			**Threshold opt. upper bound** **=** **0.15**	**MRSA (332/4446** **=** **7.47%)**			**Threshold opt. upper bound** **=** **0.4**	**MDRO (792/4503** **=** **17.59%)**			
	**Log**.	**XGBoost**	**Rand**.		**Log**.	**XGBoost**	**Rand**.		**Log**.	**XGBoost**	**Rand**.		**Log**.	**XGBoost**	**Rand**.	**Dec**.
	**Reg**.		**Forest**		**Reg**.		**Forest**		**Reg**.		**Forest**		**Reg**.		**Forest**	**Tree**
Training AUC	0.76	0.77	0.77	Training AUC	0.70	0.76	0.79	Training AUC	0.65	0.66	0.67	Training AUC	0.72	0.86	0.87	0.76
Testing AUC	0.80	0.77	0.77	Testing AUC	0.78	0.72	0.71	Testing AUC	0.66	0.66	0.69	Testing AUC	0.70	0.87	0.89	0.79
Testing sensitivity	0.66	0.63	0.75	Testing sensitivity	0.73	0.36	0.64	Testing sensitivity	0.48	0.56	0.71	Testing sensitivity	0.57	0.69	0.82	0.79
Testing specificity	0.76	0.75	0.66	Testing specificity	0.73	0.89	0.67	Testing specificity	0.75	0.71	0.58	Testing specificity	0.73	0.86	0.83	0.64
**Threshold opt. upper bound** **=** **0.3**	**VRE (503/3860** **=** **13.03%)**			**Threshold opt. upper bound** **=** **0.030**	**CRE (53/3661** **=** **1.45%)**			**Threshold opt. upper bound** **=** **0.2**	**MRSA (332/4446** **=** **7.47%)**			**Threshold opt. upper bound** **=** **0.5**	**MDRO (792/4503** **=** **17.59%)**			
	**Log**.	**XGBoost**	**Rand**.		**Log**.	**XGBoost**	**Rand**.		**Log**.	**XGBoost**	**Rand**.		**Log**.	**XGBoost**	**Rand**.	**Dec**.
	**Reg**.		**Forest**		**Reg**.		**Forest**		**Reg**.		**Forest**		**Reg**.		**Forest**	**Tree**
Training AUC	0.76	0.77	0.77	Training AUC	0.70	0.76	0.79	Training AUC	0.65	0.66	0.66	Training AUC	0.72	0.86	0.88	0.76
Testing AUC	0.80	0.77	0.77	Testing AUC	0.78	0.72	0.71	Testing AUC	0.66	0.66	0.69	Testing AUC	0.70	0.87	0.89	0.80
Testing sensitivity	0.66	0.65	0.61	Testing sensitivity	0.64	0.36	0.64	Testing sensitivity	0.70	0.45	0.67	Testing sensitivity	0.56	0.70	0.85	0.77
Testing specificity	0.78	0.72	0.74	Testing specificity	0.78	0.91	0.74	Testing specificity	0.55	0.78	0.59	Testing specificity	0.75	0.84	0.79	0.70
**Threshold opt. upper bound** **=** **0.5**	**VRE (503/3860** **=** **13.03%)**			**Threshold opt. upper bound** **=** **0.050**	**CRE (53/3661** **=** **1.45%)**			**Threshold opt. upper bound** **=** **0.3**	**MRSA (332/4446** **=** **7.47%)**			**Threshold opt. upper bound** **=** **0.6**	**MDRO (792/4503** **=** **17.59%)**			
	**Log**.	**XGBoost**	**Rand**.		**Log**.	**XGBoost**	**Rand**.		**Log**.	**XGBoost**	**Rand**.		**Log**.	**XGBoost**	**Rand**.	**Dec**.
	**Reg**.		**Forest**		**Reg**.		**Forest**		**Reg**.		**Forest**		**Reg**.		**Forest**	**Tree**
Training AUC	0.76	0.77	0.77	Training AUC	0.70	0.76	0.79	Training AUC	0.65	0.65	0.66	Training AUC	0.72	0.86	0.88	0.77
Testing AUC	0.80	0.77	0.78	Testing AUC	0.78	0.72	0.72	Testing AUC	0.66	0.66	0.70	Testing AUC	0.70	0.87	0.89	0.80
Testing sensitivity	0.61	0.59	0.63	Testing sensitivity	0.55	0.27	0.64	Testing sensitivity	0.48	0.24	0.76	Testing sensitivity	0.58	0.70	0.84	0.89
Testing specificity	0.82	0.77	0.74	Testing specificity	0.82	0.94	0.79	Testing specificity	0.75	0.89	0.59	Testing specificity	0.72	0.85	0.79	0.62

After considering all of the models that we trained for each outcome, we selected the ones with the highest (out-of-sample) Youden index, which we summarize in Table 5. For VRE, the best performing model generated a Youden index of 0.46, achieved *via* the LR model. By comparison, the RF and XGBoost models generated Youden index values of 0.41 and 0.39, respectively. For CRE, the XGBoost algorithm generate the highest Youden index (0.50), followed by LR (0.45) and RF (0.42). The performance for MRSA was noticeably lower than the other outcomes, for which RF achieved the highest Youden index (0.34). Finally, the prediction models for the aggregate MDRO outcome produced the highest Youden index values when compared to the individual MDRO outcomes, with the RF model (0.65) outperforming the XGBoost (0.57) and LR models (0.30). We note here that the tree-based models performed significantly better than the linear LR model for this aggregated outcome, which was likely due to the former's natural ability to capture nonlinear and complex interactions. In an effort to provide support for this hypothesis, we also tested the performance of a single classification tree (80) (0.54), which also performed significantly better than the LR model for this particular outcome. On the other hand, for separate VRE, CRE, and MRSA predictions, the single tree models were always dominated by (at least one of) the other algorithms, and hence, not presented in Table 4.

**Table 5:** Performance summary of the supervised machine learning models with the highest Youden's index.

**Models with the** **best Youden index**	**VRE (503/3860 = 13.03%)**			**Models with the** **best Youden index**	**MRSA (332/4446 = 7.47%)**			
	**Log. Reg**.	**XGBoost**	**Rand. Forest**		**Log. Reg**.	**XGBoost**	**Rand. Forest**	
Training AUC	0.76	0.77	0.77	Training AUC	0.65	0.66	0.66	
Testing AUC	0.80	0.77	0.77	Testing AUC	0.66	0.66	0.70	
Testing sensitivity	0.80	0.73	0.75	Testing sensitivity	0.70	0.67	0.76	
Testing specificity	0.66	0.65	0.66	Testing specificity	0.55	0.60	0.59	
Youden index	0.46	0.39	0.41	Youden index	0.24	0.27	0.34	
Threshold opt. bound	0.15	0.15	0.20	Threshold opt. bound	0.20	0.10	0.30	
**Models with the** **best Youden index**	**CRE (53/3661** **=** **1.45%)**			**Models with the** **best Youden index**	**MDRO (792/4503** **=** **17.59%)**			
	**Log. Reg**.	**XGBoost**	**Rand. Forest**		**Log. Reg**.	**XGBoost**	**Rand. Forest**	**Dec. Tree**
Training AUC	0.70	0.76	0.79	Training AUC	0.72	0.86	0.87	0.76
Testing AUC	0.78	0.72	0.72	Testing AUC	0.70	0.87	0.89	0.81
Testing sensitivity	0.73	0.73	0.64	Testing sensitivity	0.56	0.75	0.82	0.89
Testing specificity	0.73	0.77	0.79	Testing specificity	0.75	0.82	0.83	0.65
Youden index	0.45	0.50	0.42	Youden index	0.30	0.57	0.65	0.54
Threshold opt. bound	0.025	0.015	0.05	Threshold opt. bound	0.50	0.30	0.40	0.30

For each model presented in Table 5, the difference between the (out-of-sample) AUC for the (cross-validated) training and testing sets were typically small, suggesting well-trained models without significant overfitting. The LR and RF models for CRE demonstrated larger gaps, suggesting that these models might be slightly less robust than others; however, this volatility is likely explained by the extremely low prevalence of positive cases on which to train the models. The best predictions for VRE colonization upon ICU admission were generated by the LR model, which achieved 80% sensitivity and 66% specificity. For CRE, XGBoost produced the best model, having 73% sensitivity and 77% specificity. For MRSA, the RF model performed best, yielding 76% sensitivity and 59% specificity. Finally, the most effective model for the aggregate MDRO outcome was a random forest model, which was capable of detecting 82% of colonized patients with 83% specificity.

In addition to generating predictions, we also used our modeling framework to identify the key predictors for separate and aggregate VRE, CRE, and MRSA colonization. In Table 6, we summarize the top five predictors for the models reported in Table 2, and provide their ranking in the corresponding models as indicated by OR and FI. See the Supplementary Material (Appendix C) for the OR and FI values of the factors presented in Table 6.

**Table 6:** Top five common predictors for VRE, CRE, MRSA, and MDRO colonization identified by the machine learning models.

**Top five common predictors for VRE colonization upon ICU admission**					
**VRE colonization upon ICU admission**		**Relative ranking**			
**Factors**	**Features**	**Log. Reg**.	**XGBoost**	**Rand. Forest**	
Long-term care facility stay	Yes	1	3	1	
Recent 1-digit ICD10 procedure	Other procedures	2	1	2	
Current diagnosis CCS class	Skin and subcutaneous tissue	3	2	3	
Recent 2-digit ICD10 procedure	Medical/surgical anatomical	6	5	8	
Recent 2-digit ICD10 procedure	Administration circulatory	8	4	6	
**Top five common predictors for CRE colonization upon ICU admission**					
**CRE Colonization upon ICU Admission**		**Relative Ranking**			
**Factors**	**Features**	**Log. Reg**.	**XGBoost**	**Rand. Forest**	
Current diagnosis CCS class	Skin and subcutaneous tissue	2	2	1	
Recent 1-digit ICD10 procedure	Other procedures	3	3	2	
Prior ICU stay	> 20 days	4	6	5	
Long-term care facility stay	Yes	5	6	6	
Number of current diagnosis PoA	> 30 and ≤ 50	6	8	3	
**Top five common predictors for MRSA colonization upon ICU admission**					
**MRSA colonization upon ICU admission**		**Relative ranking**			
**Factors**	**Features**	**Log. Reg**.	**XGBoost**	**Rand. Forest**	
Recent 1-digit ICD10 procedure	Other procedures	1	2	1	
Current diagnosis CCS class	Skin and subcutaneous tissue	2	9	2	
Current diagnosis CCS class	Injury and poisoning	7	1	8	
Current diagnosis CCS class	Infectious and parasitic	9	8	5	
Recent 1-digit ICD10 procedure	Administration	−3	3	14	
**Top five common predictors for MDRO colonization upon ICU admission**					
**MDRO colonization upon ICU admission**		**Relative ranking**			
**Factors**	**Features**	**Log. Reg**.	**XGBoost**	**Rand. Forest**	**Dec. Tree**
Recent 1-digit ICD10 procedure	Other procedures	7	1	1	2
Current diagnosis CCS class	Skin and subcutaneous tissue	16	2	2	14
Current diagnosis CCS class	Mental illness	35	6	3	12
Current diagnosis CCS class	Infectious and parasitic	57	12	4	16
Sex	Female	89	3	5	9

Among the recent ICD-10 procedures that were performed during the current hospital stay before ICU admission, the procedures categorized as “Other Procedures” in the ICD-10 PCS were among the top five predictors for VRE, CRE, MRSA, and MDRO. In our dataset, a significant proportion of these procedures were “8E0ZXY6”, an ICD-10 code designated for isolation precautions. The patients having a history of a prior colonization or infection for a given MDRO (or are at risk for another indication) were flagged with this code upon admission to the hospital so that they were closely monitored (and if needed, isolated) during their hospital stay. Our results presented in Table 6 show that these patients were at a higher risk for being colonized with an MDRO at ICU admission regardless of the specific indication for which the close monitoring and isolation precautions were put in place.

Another key predictor for VRE, CRE, MRSA, and MDRO colonization is the CCS-based diagnosis category “skin and subcutaneous tissue disease” that was PoA (Table 6). The diagnoses that fall under this CCS category were determined for the current hospital admission and included rash, cellulitis, cutaneous abscess, pressure ulcer, non-pressure chronic ulcer, and other skin conditions. Our finding resonates with the clinical literature and practice, as skin and soft tissue infections are amongst the most common bacterial infections, are mostly treated with antibiotics that might cause antimicrobial resistance (81). Further, skin and soft tissue infections are the most frequently reported clinical manifestations of community-acquired MRSA (82).

For MDRO and in particular MRSA, the CCS-based current diagnosis category “infectious and parasitic diseases” was one of the critical factors that increase the risk of colonization. This category included diseases such as chronic viral hepatitis C, bacteremia, human immunodeficiency virus (HIV), and sepsis. Patients with these diseases might be at higher risk for MDRO, and in particular MRSA, colonization due to a compromised immune system.

For VRE and CRE, having a prior long-term care facility (LTCF) stay was one of the key predictors for colonization upon ICU admission. This association between VRE or CRE colonization and a previous LTCF stay has been reported by other studies (83, 84) (also see the Supplementary Material Appendix B). High rates of MDRO colonization, debilitating diseases, and the receipt of multiple antibiotics among LTCF residents are likely to be the primary causes of this association both for VRE and CRE colonization (85).

Other key predictors for VRE were recent procedures “administration circulatory” (ICD-10-PCS ‘30'), such as transfusion, and “medical and surgical anatomical regions, general” (‘0W'), such as drainage, insertion, removal, and transplantation procedures. For CRE, a prior ICU stay longer than 20 days and a total number of diagnoses PoA (i.e., current diagnoses) >30 were two critical factors increasing the risk of colonization. For MRSA, the current diagnosis for “injury and poisoning”, mostly consisting of procedural injuries such as accidental puncture or dural laceration during a procedure, is associated with an increased colonization risk. On the contrary, the recent procedure code for “administration” (i.e., ICD-10 PCS codes with first character “3”) was found to lower the risk of colonization. Finally, female sex and the “mental illness” category for current diagnosis, including diagnosis for cocaine abuse, opioid abuse, poisoning by heroin and psychological disorders, were two other key factors associated with an increased risk for MDRO colonization. Patients in this category (i.e., the “mental illness”) are at higher risk for using injections and causing damage to their skin, which might explain the increased risk for MDRO colonization.

## Discussions

Leveraging a rich dataset and supervised ML algorithms, we developed an accurate and interpretable framework for predicting VRE, CRE, and MRSA colonization upon ICU admission. The developed predictive analytics framework achieved the following sensitivity and specificity values for VRE, CRE, and MRSA colonization: 80% and 66% for VRE with LR, 73% and 77% for CRE with XGBoost, and 76% and 59% for MRSA with RF. Further, we predicted MDRO (i.e., VRE, CRE, or MRSA) colonization as an aggregate outcome with 82% sensitivity and 83% specificity for MDRO using RF.

These results indicate that predicting MDRO colonization in aggregate, rather than separately predicting VRE, CRE, and MRSA, achieved the highest prediction accuracy in terms of both AUC and Youden's index. On the one hand, predicting a specific MDRO would be preferable, as it would enable more customized interventions such as tailored antibiotic therapy. On the other hand, accurately predicting MDRO colonization without specifying whether it is VRE, CRE, or MRSA is still quite important for clinical practice. This is because the key interventions for these MDROs are the same or similar, such as contact precautions and enhanced environmental cleaning, and can later be followed up by more specific testing protocols to identify the underlying organism. Accordingly, many infection control measures can be implemented rapidly upon ICU admission for the patients who are suspected to be colonized, and treatment strategies and more advanced interventions can be tailored later as more information becomes available.

In addition to producing timely predictions for newly admitted ICU patients, our ML-based modeling framework can also be utilized to identify the key predictors for VRE, CRE, and MRSA colonization upon ICU admission. We identified several important predictors of MDRO colonization, including long-term care facility exposure, a current diagnosis of skin/subcutaneous tissue or infectious/parasitic disease, and a recent ICD-10 procedure “Other Procedures”, including isolation precaution procedures, as the key predictors for MDRO colonization upon ICU admission. These predictors can help characterize and identify ICU patients at high-risk for MDRO colonization and hence, facilitate timely implementation of infection control measures such as selective use of contact precautions, targeted surveillance, and tailored antibiotic therapy.

The primary limitation of our study was that we did not utilize any data on patient medical history outside of UMMC. For example, we did not take into account antibiotic consumption outside of UMMC or during outpatient visits. Similarly, we did not have information about patients who could have been admitted elsewhere, thus censoring any information about whether they received or underwent additional treatments and procedures in other healthcare facilities. As we utilized administrative data for procedures and diagnoses, which are primarily used for billing, we did not have full access to exact clinical conditions and we did not know the exact reason why a specific procedure was performed or diagnosis was established. Our discussions with clinicians shed some light on these uncertainties but we could not determine the exact details for each individual patient other than what the data conveys. Finally, our data were derived from a single source and we were only able to observe the performance of our modeling framework on an out-of-sample subset from the same facility.

The machine learning algorithms we used in this study had additional limitations. Specifically, logistic regression models assume predictors to have a linear relationship with the log odds (i.e., the logit form) of the prediction variable and may have difficulty in capturing complex non-linear relations. Furthermore, in their standard forms, logistic regression models require minimal or no multicollinearity between independent variables, and hence, the presence of highly correlated predictors might be problematic. Overfitting might also be a significant issue for the logistic regression algorithm but this can be avoided by the use of a regularization technique. XGBoost (i.e., eXtreme Gradient Boosting) can also easily overfit if its parameters are not tuned properly. Further, like any other boosting method, XGBoost models are quite sensitive to outliers since the XGBoost method relies on the sequential ensemble of decision trees and every decision tree classifier attempts to fix the errors of its predecessor learners. Finally, assuming no formal parametric structure or distribution and relying on the parallel ensemble of decision trees, random forest models can cope with skewed data and can capture complex non-linear relationship. Yet, using a random forest algorithm with the default values can also generate suboptimal results (86), and hence, parameter and hyperparameters tuning should be performed to increase model performance. Moreover, generated feature importance scores, demonstrating the relevant importance of each feature for prediction, are not sufficient to capture all forms of dependencies between predictors and prediction outcome. Partial dependence plots have been recommended to be used to address this shortcoming (86). Last but not least, random forest models are biased in favor of categorical predictors having noticeably more levels and hence, general conclusions solely based on feature importance scores might not always be reliable.

It is noteworthy to emphasize that our study, which focused on predicting MDRO colonization for newly admitted ICU patients, would not prevent the importation of VRE, CRE, and MRSA into the ICU setting. However, by producing reliable predictions and identifying key risk factors for colonization, our approach could enable early detection of colonized patients and facilitate timely and targeted implementation of preventive measures on asymptomatic MDRO carriers. That is, once implemented as a clinical decision support system, our predictive analytics framework could alert healthcare providers in real-time when a high-risk patient, characterized by the predictors identified by this study, is admitted to the ICU so that the medical team can apply the necessary precautions, such as contact precautions, in a timely manner to prevent potential transmissions. This approach could help reduce transmission of these so-called “superbugs” in ICUs, and would particularly be useful for healthcare settings where active surveillance is not performed. In future efforts, we plan to examine the practical utility of our modeling framework *via* a comprehensive computational simulation study that investigates and quantifies the estimated value of early detections flagged by our model both in hospital and region settings by separately using agent-based and network-based simulation models (87).

Several recent studies also proposed or assessed a predictive modeling approach for MDROs. Studying MDRO infections in emergency department settings, González del Castillo et al. (88) proposed a prediction model, developed by using backward logistic regression. The model achieved an AUC of 0.76 and 0.72 in the model training and testing sets, respectively. Splitting patients into six risk categories, the authors also examined different cut-off values for the risk scores. The model with the optimal cut-off value achieved 59% sensitivity and 74% specificity. Faine et al. (89) performed an external validation study to test the performance of the predictive clinical decision rule they previously developed *via* logistic regression to identify multidrug-resistant urinary pathogens in the emergency department. The model yielded a sensitivity of 56% and specificity of 66% in the validation cohort. Tseng et al. (90) utilized a multivariate logistic regression to develop a statistical model for predicting multidrug-resistant gram-negative bacteria colonization and infections at the time of hospital admission. The AUC values of their model were 0.75 and 0.80 in the model development and validation sets, respectively. The authors also identified the best threshold value maximizing the Youden index with 57% sensitivity and 85% specificity. Goodman et al. (91) derived and compared a ML-based decision tree (i.e., classification and regression tree) with a logistic regression-derived risk score for *extended-spectrum beta-lactamase* (ESBL) bacterial infections. The sensitivity and specificity values of the classification and regression tree (CART) were 51.0 and 99.1%, respectively. The AUC was 0.77 for the CART model, 0.87 for the multivariable LR model, and 0.87 (and 0.89 following cross-validation) for the LR-based risk score. The risk score achieved a sensitivity of 49.5% and a specificity of 99.5% with the cutoff value that maximizes the overall ESBL classification accuracy. Sullivan et al. (92) developed a regression model to predict carbapenem resistance among patients with *Klebsiella pneumoniae bacteremia*. The mean AUC of the model was 0.73, which achieved 73% sensitivity and 59% specificity in the testing set. Lee et al. (93) assessed the performance of an artificial neural network (ANN)-based prediction model for predicting bacteremia in comparison with naïve Bayesian, support vector machine (SVM), and RF models. Among the compared models, the multi-layer perceptron, a feedforward ANN model, the authors developed exhibited the highest sensitivity (81%) and had a specificity rate 59% with an AUC 0.73. Finally, Lewin-Epstein et al. (94) applied several ML algorithms, consisting of LR with LASSO, neural networks, gradient boosted trees, and an ensemble of these three ML algorithms, to predict antibiotic resistance profiles of bacterial infections among hospitalized patients. The ensemble model achieved AUC values ranging from 0.73 and 0.79 for different types of antibiotics, which were improved to 0.80–0.88 if the infecting bacterial species was assumed to be known. As a comparison with these studies, the best performing model in our study (RF for MDRO prediction) achieved 0.87 and 0.89 AUC in training and testing sets, respectively, and yielded 82% sensitivity and 83% specificity in the validation/testing cohort. In general, the use of tree-based ensemble algorithms, such as XGBoost and random forest, played an important role in achieving higher predictive accuracy in our study.

Prediction models have been previously reported to perform worse when they are implemented in clinical practice and applied to new individuals that are different than the original study population that the model was derived (95). Therefore, before being integrated into practice for clinical decision support, the robustness of the proposed approach must be thoroughly examined and externally validated in different populations. To address this critical concern, we are currently studying the transportability, generalizability, and external validation of our ML models and predictive analytics framework by leveraging retrospective EHR data from another academic teaching hospital, located in Baltimore, Maryland, USA. We plan to publish the findings of this ongoing study in a separate article.

Traditionally, many prediction rules, developed as a decision support tool for clinicians, are designed to be very simple, relying on only a small number of variables, for practicality. Yet, with the increasing availability of electronic healthcare record data and the expansion of modern database and software systems, the use of data-driven prediction models and other analytical and computational methods for the identification, control, and prevention of MDROs and other HAIs has been increasing (56). As a result, a growing number of healthcare facilities are capable of generating more complex prediction models in an automated fashion. Accordingly, taking advantage of the advances in computational and data recording technologies, many healthcare organizations can use our data-driven prediction framework to produce real-time predictions and identify the high-risk patients for MDRO colonization.

Finally, we touch upon the topic of the general trade-off between the predictive power of ML algorithms and the interpretability of ML models and their results. This trade-off derives from the fact that the best performing algorithms are often the most complex ones. That is, while simpler models such as regressions and decision trees, are transparent and explainable by design, more advanced models that can capture and cope with higher levels of complexities (e.g., neural network, random forest, XGBoost) are typically more complex and of “black-box” nature (96). Clinicians are more accustomed to simpler traditional models (e.g., logistic regression), as these models usually provide better understanding for the reasoning chain behind the predictions made. Therefore, we summarize the odds ratios of the best performing LR models in Table 7, separately for CRE, VRE, and MRSA colonization. As known, an odds ratio value > 1 indicates positive correlation whereas an odds ratio value <1 means that the presence of the corresponding feature reduces the risk of colonization. We note that the best performing LR models are not necessarily the best performing ML models but their outputs (i.e., the odds ratios for each feature) offer an easier interpretation of the results.

**Table 7:** Predictors and coefficients (i.e., odds ratios) of the best performing logistic regression models.

**Predictor/factor**	**Categorical level**	**CRE**	**VRE**	**MRSA**
Prior diagnosis CCS class	Neoplasms	2.00	-	-
	Blood and blood-forming organs	-	1.36	-
	Infectious and parasitic	-	1.18	-
	Mental illness	0.84	-	-
	Symptoms, signs, ill-defined conditions	0.79	-	-
	Circulatory system	-	-	0.81
Current diagnosis CCS class	Skin and subcutaneous tissue	1.92	1.55	1.52
	Nervous system and sense organs			1.26
	Respiratory system			1.25
	Injury and poisoning			1.15
	Infectious and parasitic			1.07
	Genitourinary system	-	1.18	-
	Mental illness		-	1.02
	Circulatory system	-	-	0.85
	Endocrine, nutritional, metabolic, immunity	0.88	-	0.65
	Neoplasms	0.75	-	0.83
Recent 1-digit ICD 10 procedure	Other procedures	1.76	1.80	1.76
	Extracorporeal/systemic	1.30	-	-
	Administration	-	-	0.68
	Medical and surgical	-	-	0.90
Prior ICU stay	> 0 Days and <5 Days	1.20	1.02	-
	10–20 Days	-	1.39	-
	> 20 Days	1.73	-	-
Prior 2-digit ICD 10 procedure	Medical/surgical gastrointestinal	1.35	-	1.05
	Medical/surgical upper veins	-	1.03	-
	Medical/surgical respiratory	-	-	1.33
Recent 2-digit ICD 10 procedure	Medical/surgical gastrointestinal	-	-	1.32
	Medical/surgical anatomical	-	1.33	-
	Medical/surgical heart and vessels	1.21	-	-
	Administration circulatory	1.27	1.28	-
	Medical/surgical hepatobiliary	-	1.13	-
Prior antibiotics use	Yes	1.25	-	-
	Prior antibiotics Fluoro use	1.28	-	-
	Prior antibiotics Ceph use	-	1.09	-
	Number of different types used = 3	1.19	-	-
Number of recent procedures	≤ 2	0.92	-	0.87
	> 2 and ≤ 5	-	0.91	-
	> 5 and ≤ 10	-	0.89	0.96
Number of prior diagnosis	≤ 10	-	-	0.91
	> 10 and ≤ 20	-	1.01	-
	> 50 and ≤ 100	-	1.04	-
Number of prior procedures	> 20	-	1.31	-
Number of current diagnosis	≤ 10	-	0.72	0.67
	> 10 and ≤ 20	0.74	0.89	-
	> 30 and ≤ 50	1.38	1.03	-
Admission type or source	Elective	0.96	0.78	-
	Home or self referral	0.73	0.83	-
	Physician referral	0.65	0.70	-
Race/ethinicity	Black	0.91	0.94	-
Sex	Female	-	-	0.89
Age group	Age 30–40	-	0.97	-
	Age 40–50	0.93	-	-
Long-term care facility stay	Yes	1.69	1.95	-

There are several other analyses that can be performed to improve the interpretability of the models and better communicate results with clinicians. One approach is to utilize the significant predictors and predicted probabilities identified and estimated by the best performing ML model and to link them with a linear regression. That is, after the predictive analytics study is performed, the modeler can fit a linear regression model to the significant predictors (i.e., the features with non-zero coefficients) to explain the predicted probabilities (i.e., MDRO colonization risks that the ML model predicts for each patient) and as a result, can provide a direct means to quantify the impact of each predictor on MDRO colonization risk. If desired, this approach can be taken a step further by developing a simple clinical decision rule based on the weights the linear regression model provides for each significant predictor (though, usually, at the expense of predictive power). Alternatively, another approach that can facilitate the interpretability of the results is to conduct a univariate sensitivity analysis, again, on the significant predictors and predicted probabilities of the best performing ML model. By taking this approach, the modeler can set the value of a single feature equal to zero (or equivalently, momentarily exclude it from the analysis) and then calculate the predicted probabilities by using the already trained ML model and all other significant predictors. The average decrease in the predicted probabilities (due to the absence of the feature of interest) can, then, be used to quantify the impact of (missing) feature on MDRO colonization risk. By doing this univariate sensitivity analysis on each and every significant feature, the modeler can again provide a numeric value quantifying the strength of the association between each predictor and the (predicted) MDRO colonization risk.

## Conclusion And Future Work

Timely detection of MDRO colonization, prevention of MDRO infections, and early implementation of counter-measures are of utmost importance to alleviate the harms and minimize the costs associated with MDROs at patient, hospital, and national levels. Following the advances in database management technologies, increased computational power of computers, and the availability of user-friendly software packages, descriptive and predictive analytics methods can now play a pivotal role for the analysis of patient data and the identification of patients with MDRO colonization. This was the primary objective of our study in this paper, which showcased the use and the practical utility of such data-driven methods to correctly predict the presence of VRE, CRE, and MRSA colonization at the time of ICU admission.

In this paper, we proposed a data-centric modeling framework to predict VRE, CRE, and MRSA colonization upon ICU admission and identify the associated risk factors. Our study achieved the highest prediction accuracy, measured by Youden's index, when VRE, CRE, and MRSA colonization were combined and predicted as an aggregate outcome. Capable of coping with significant class imbalance, a feature commonly observed in clinical datasets, the framework described in this study can be used as a clinical decision support tool to provide accurate on-time predictions especially if it is regularly updated and trained off-line as additional (i.e., more recent) data become available. This predictive analytics approach can further be used to identify the key risk factors and define high-risk populations, for which targeted interventions can be implemented rapidly to reduce transmission of MDROs in ICUs.

There are three research directions that we plan to pursue in near future: First, we will study the acquisition outcomes, where we focus on the ICU patients who were initially colonization-free but acquired VRE, CRE, or MRSA colonization during their ICU stay. Second, we will develop a comprehensive agent-based simulation model to analyze MDRO colonization and infection in ICUs and assess the impact of commonly utilized prevention and control measures on MDRO transmission. Finally, we are in the process of acquiring more data from another major healthcare facility to conduct a similar study by leveraging this additional dataset. This will not only enable us to enlarge the size our dataset, leading to more accurate predictions, but will also give us an opportunity to assess the generalizability of our findings and help us develop more robust predictions.

## Data Availability Statement

Data cannot be shared publicly because of private ownership. Data were obtained *via* electronic healthcare records from the University of Maryland Medical Center (UMMC), an academic teaching hospital located in Baltimore, Maryland, United States of America. Requests to access the datasets should be directed to LP, lpineles@som.umaryland.edu.

## Author Contributions

ÇÇ and SB designed the analytical modeling framework and performed verification and validation analysis. ÇÇ performed data and statistical analyses, developed the machine learning models, conducted the predictive analytics study, and generated the numerical results under the supervision of SB and wrote the first draft of the manuscript. LP coordinated the data retrieval efforts from the University of Maryland Medical Center (UMMC). EK managed the overall project. EK, LP, and AH served as subject-matter experts. SB, EK, and AH supervised the project and provided mentorship. EK, SB, LP, and AH wrote the grant proposal for funding. All authors contributed to conception, design of the study, performed major edits on the manuscript, contributed to manuscript revision, and approved the submitted version.

## Funding

This study was supported by the U.S. Centers for Disease Control and Prevention (CDC) Modeling Infectious Disease (MInD) Network under award numbers 1U01CK000536 and 5U01CK000589.

## Author Disclaimer

The content is solely the responsibility of the authors and does not necessarily represent the official views of the CDC.

## Conflict of Interest

The authors declare that the research was conducted in the absence of any commercial or financial relationships that could be construed as a potential conflict of interest.

## Publisher's Note

All claims expressed in this article are solely those of the authors and do not necessarily represent those of their affiliated organizations, or those of the publisher, the editors and the reviewers. Any product that may be evaluated in this article, or claim that may be made by its manufacturer, is not guaranteed or endorsed by the publisher.
