# Supplementary material for: A Data-Driven Framework for Identifying Intensive Care Unit Admissions Colonized With Multidrug-Resistant Organisms
Source: Front Public Health. 2022 Mar 17;10:853757. doi: 10.3389/fpubh.2022.853757 (PMC8968755; doi:10.3389/fpubh.2022.853757)
Supplement: Supplementary file 1 [file Data_Sheet_1.docx]

**Appendix A: Descriptive Statistics**

**
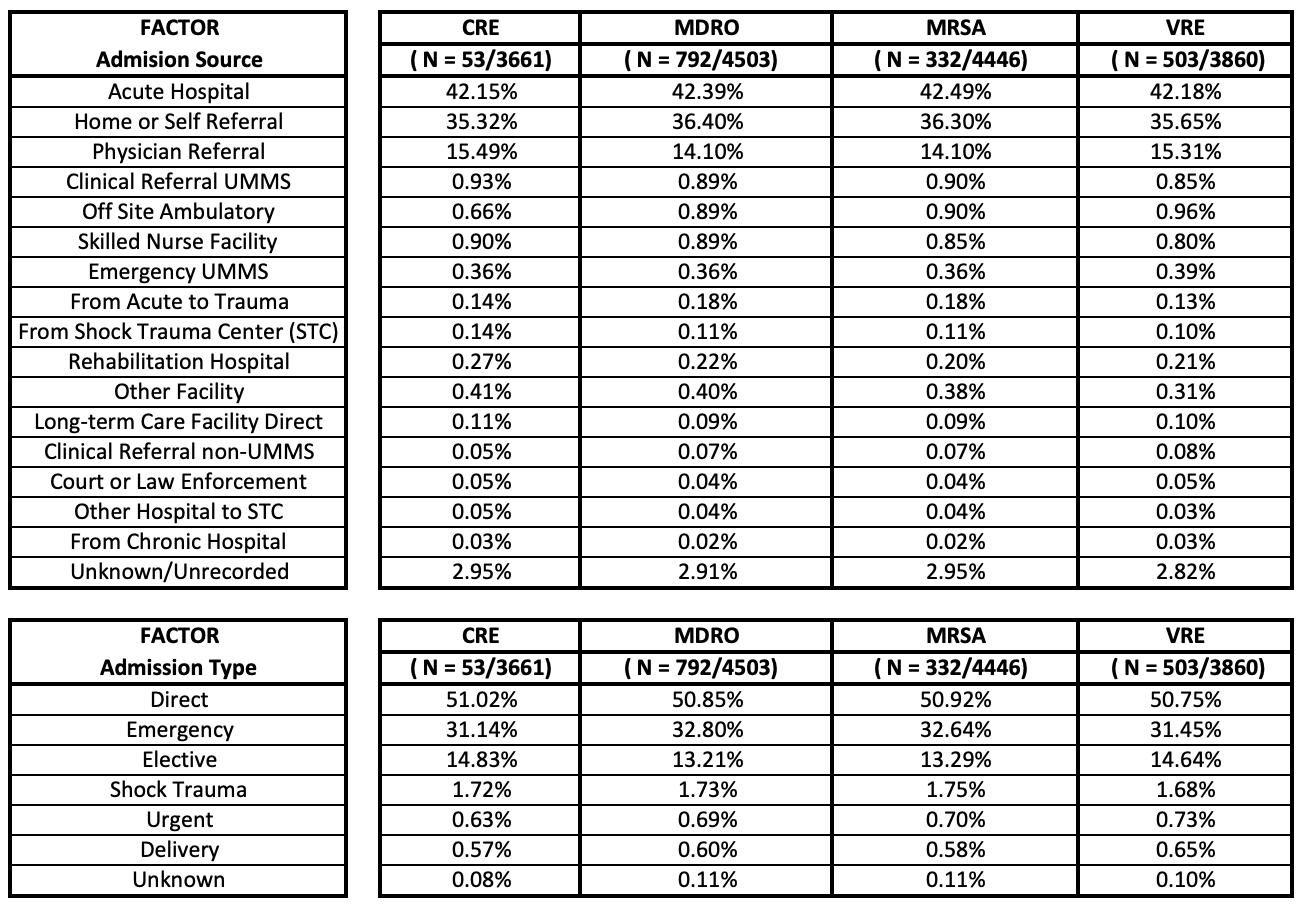
**

**
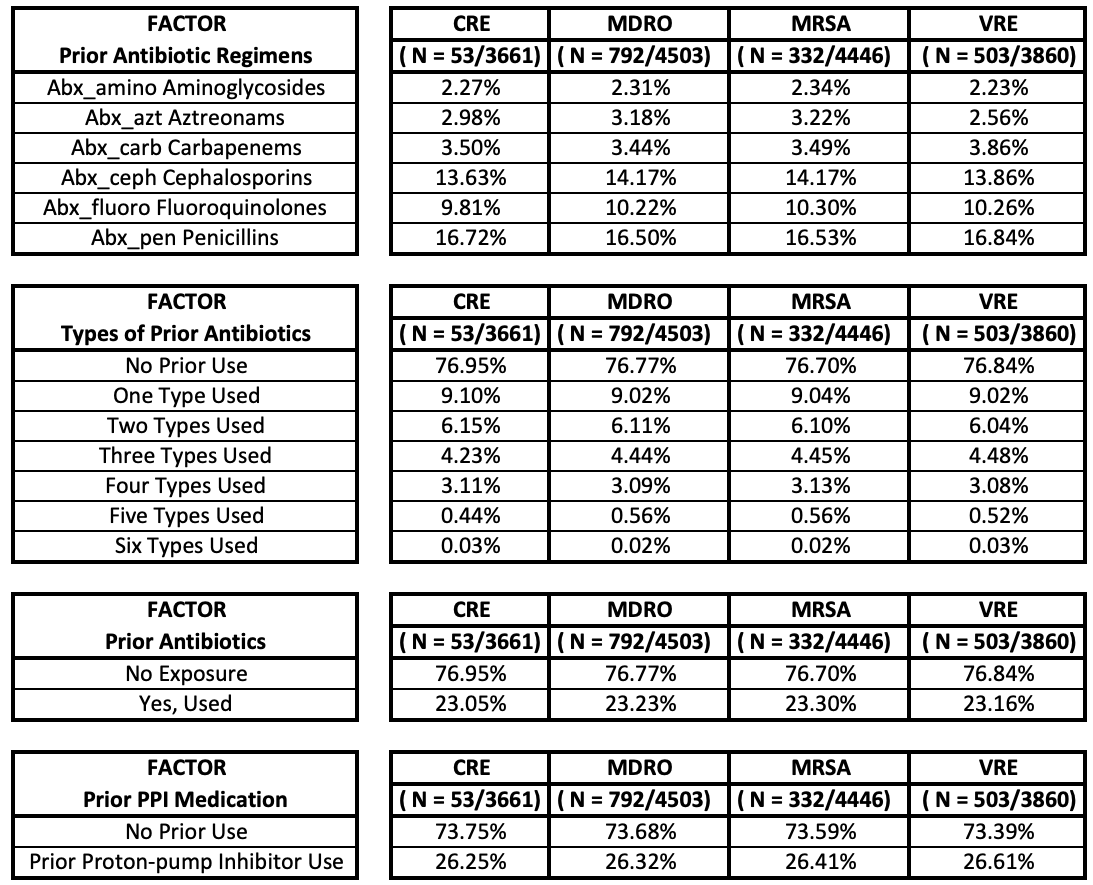
**

**
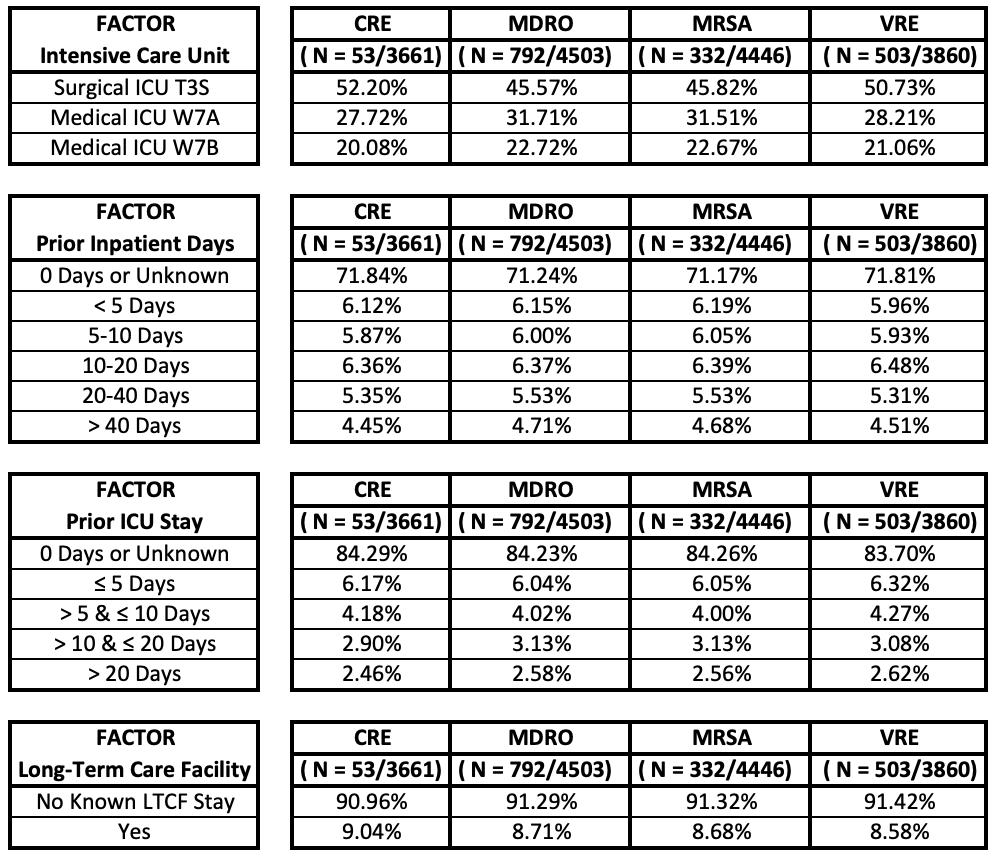
**

**
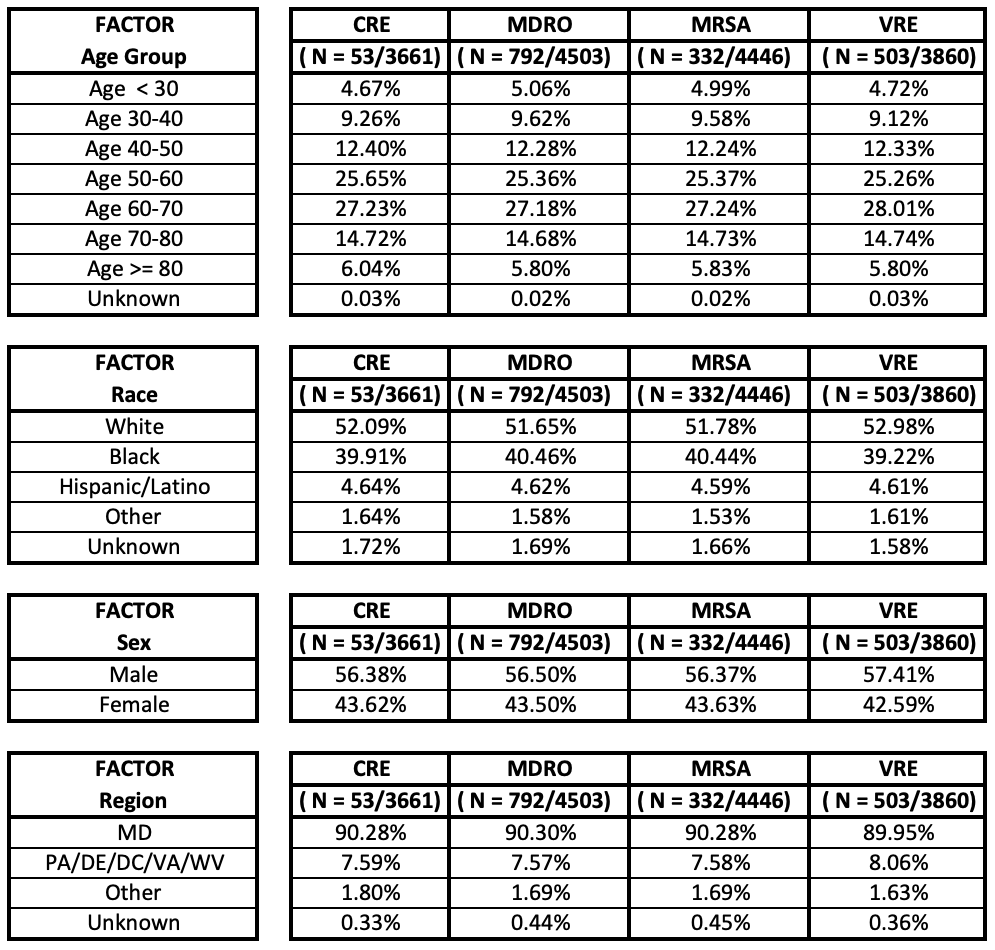
**

**
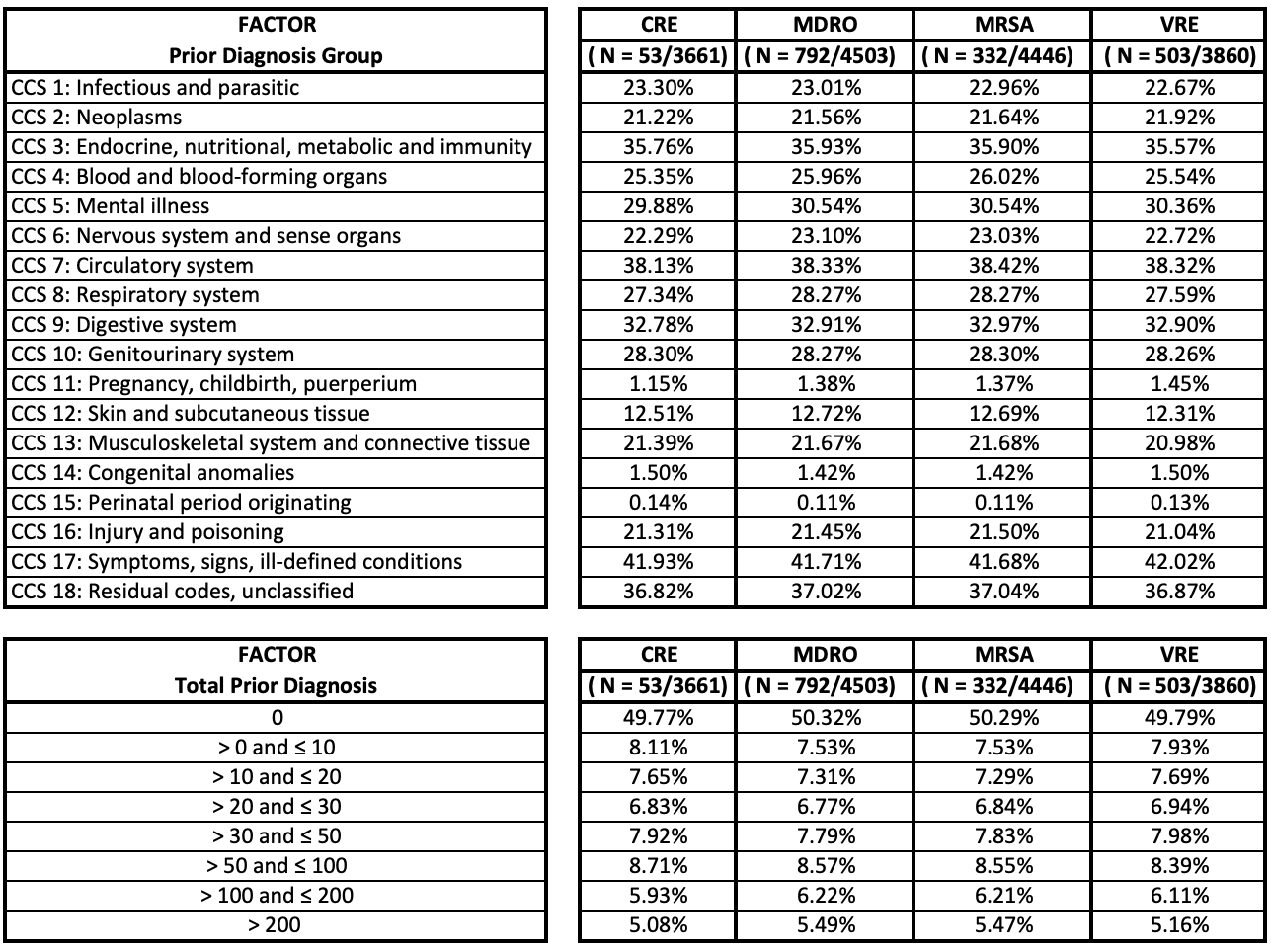
**

**
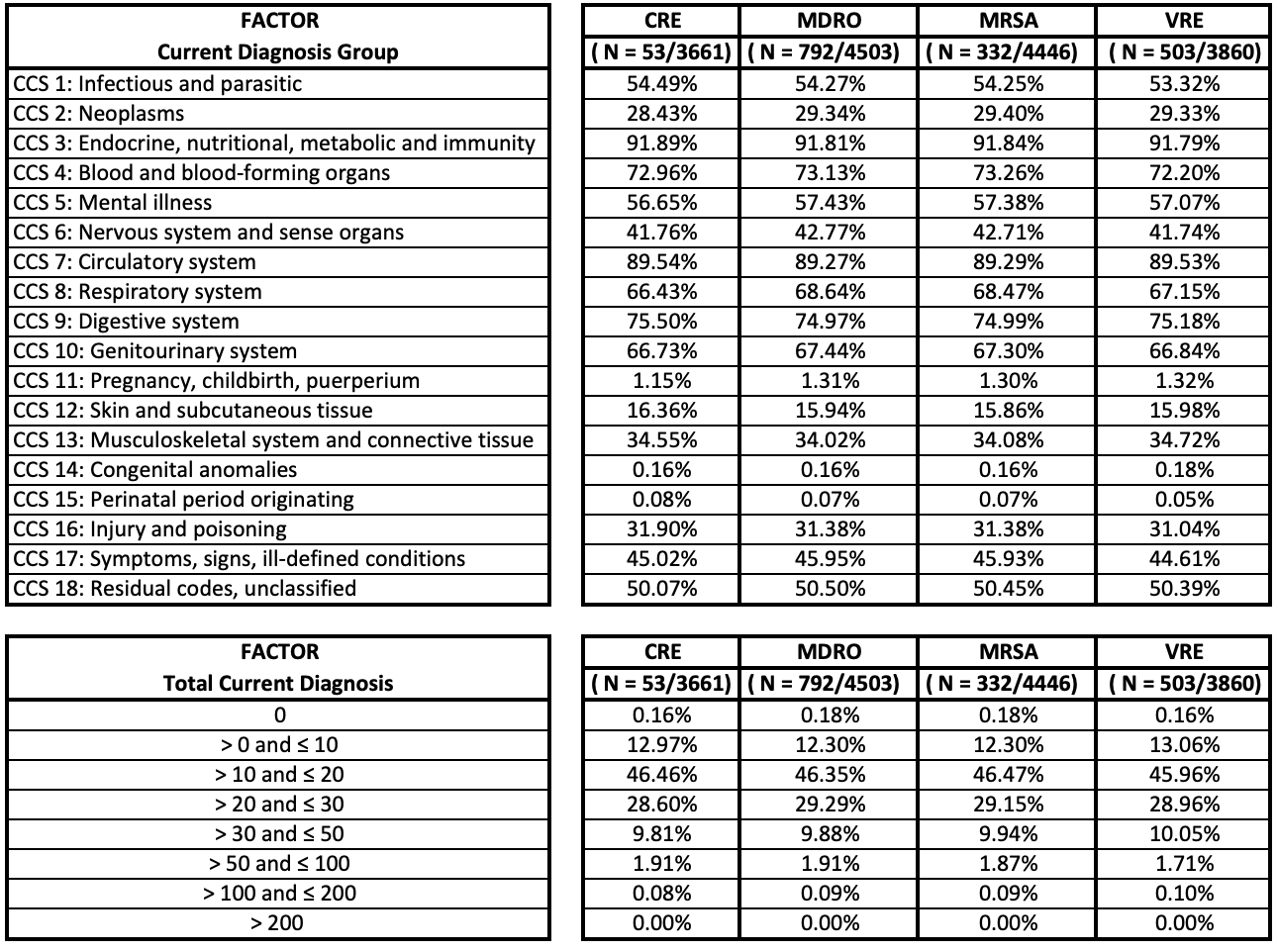
**

**
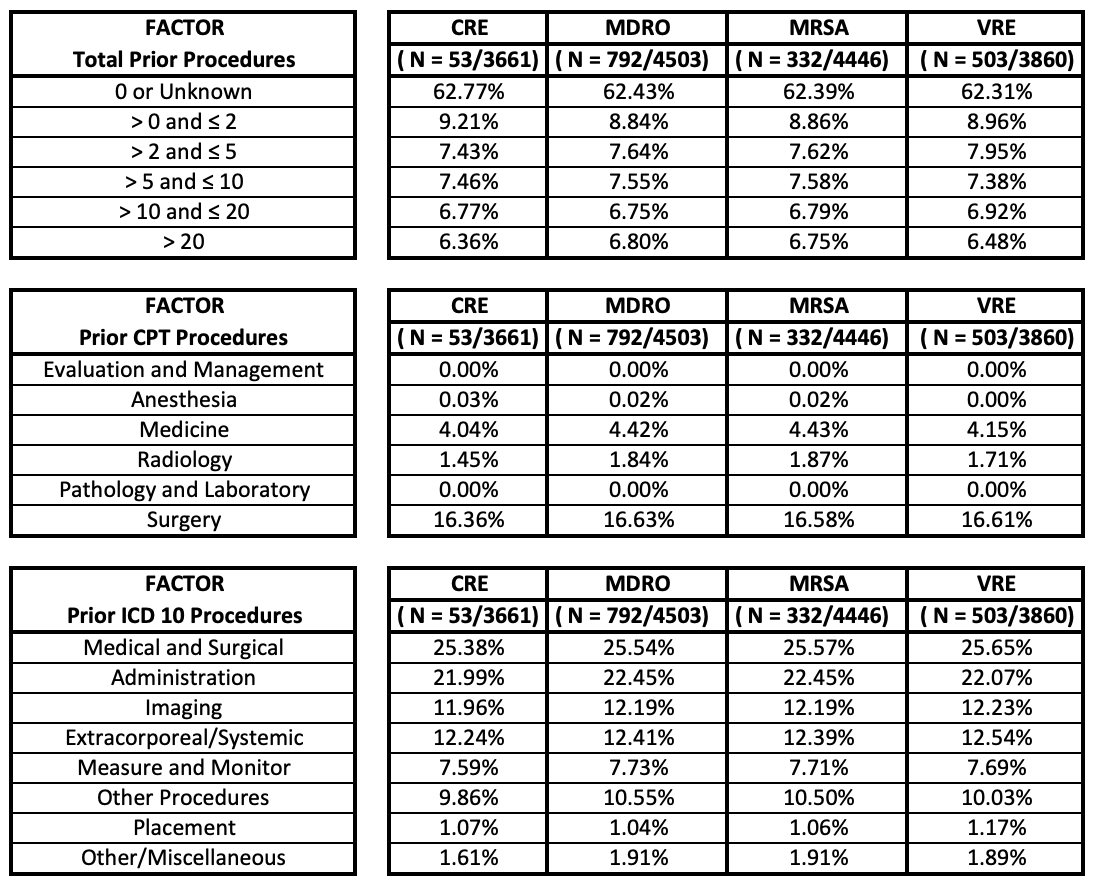
**

**
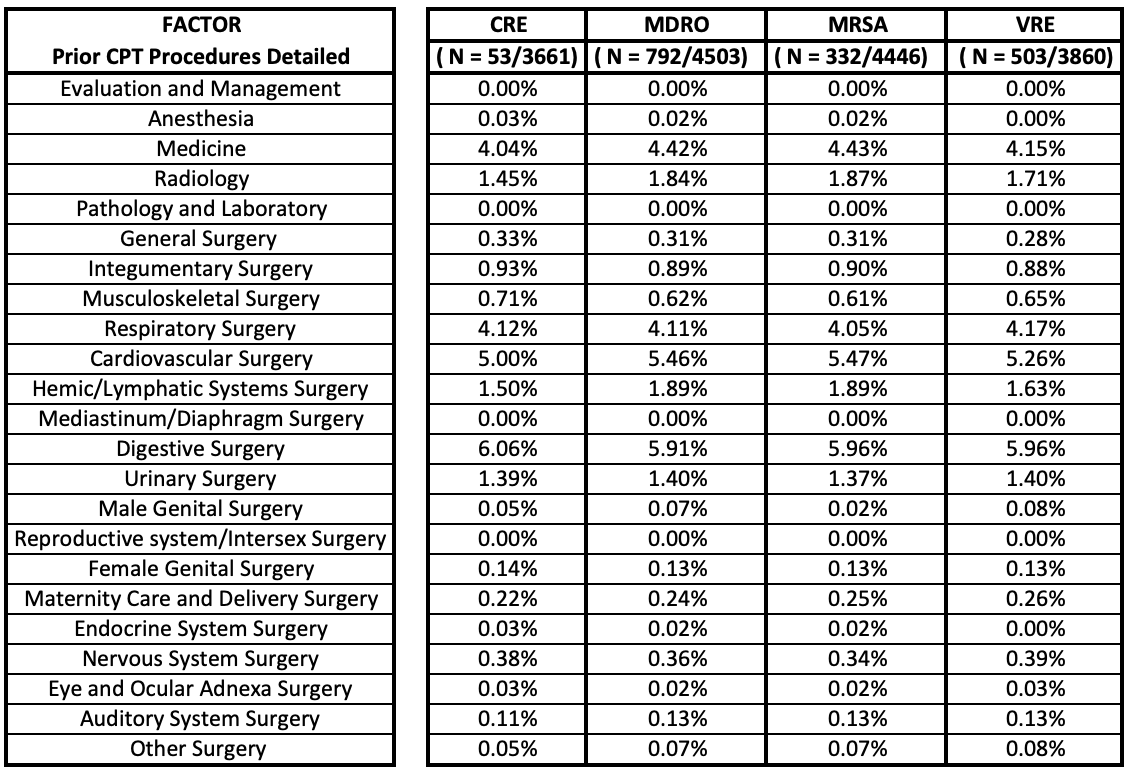
**

**
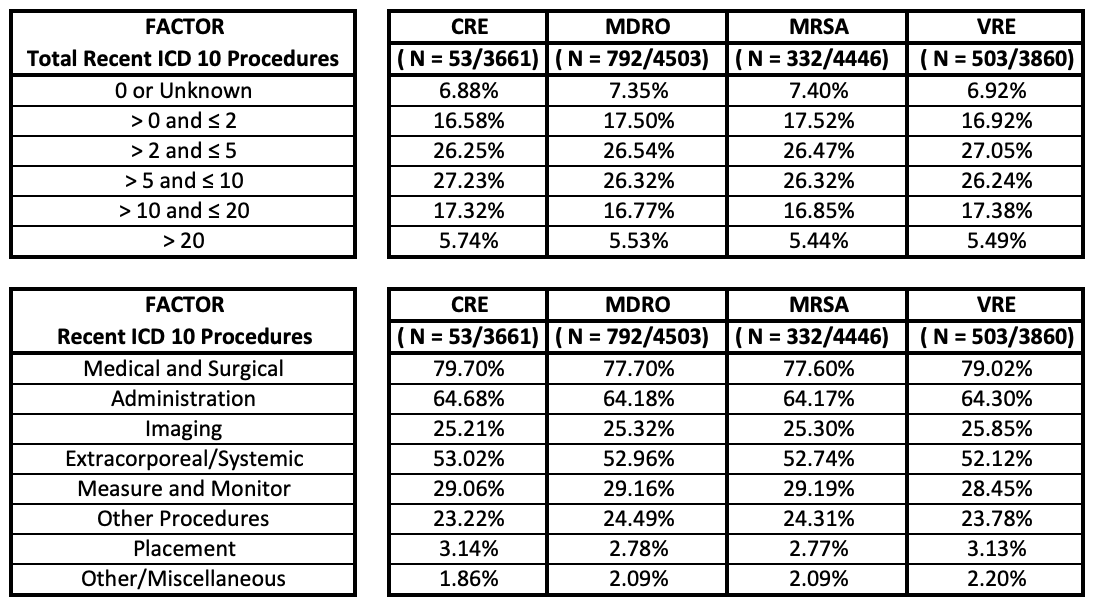
**

**
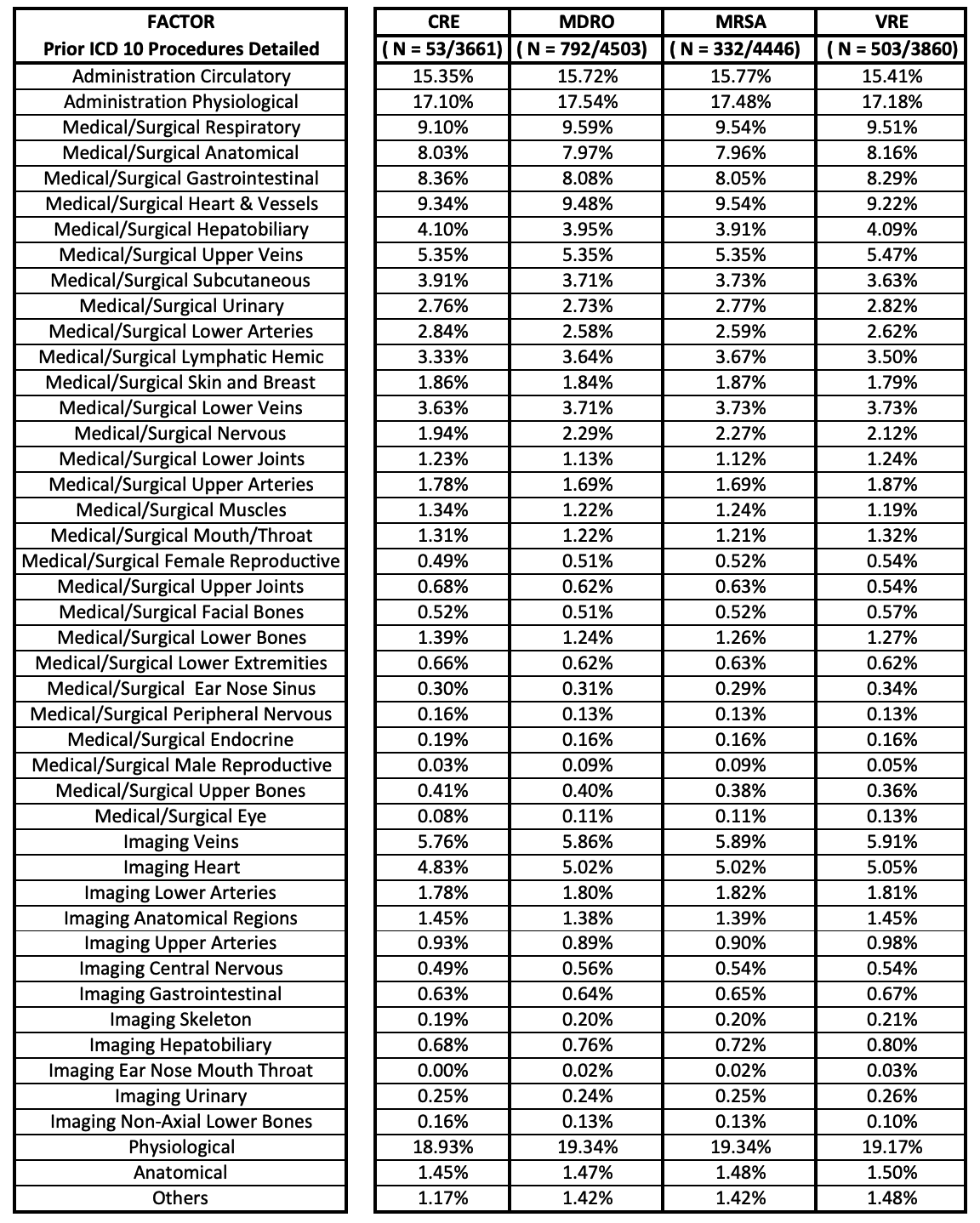
**

**
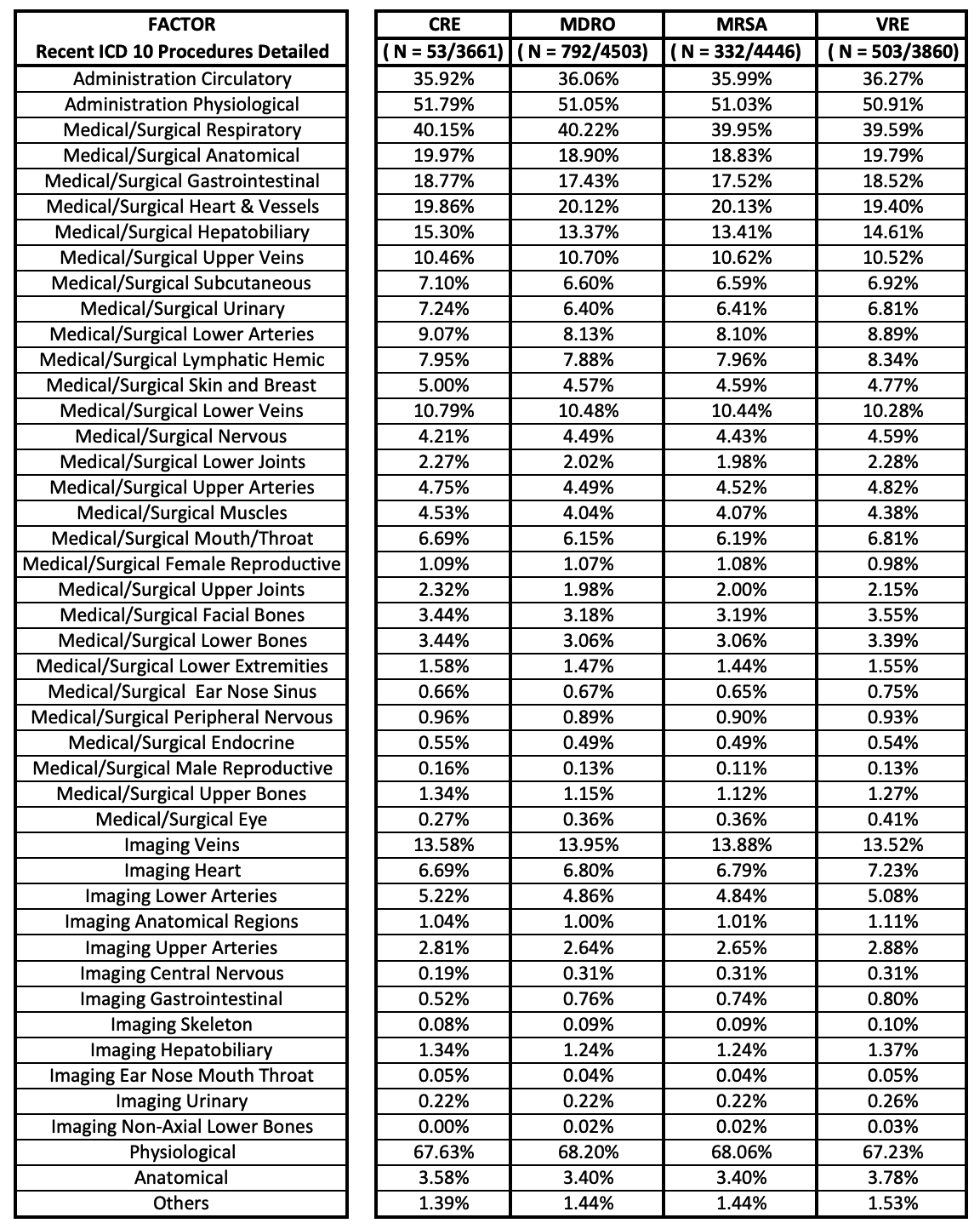
**

**Appendix B: Literature Review of Clinical Papers**

In this section, we provide a brief overview of the existing studies that (1) assess the risk factors associated with VRE, CRE and MRSA colonization, and (2) develop simple clinical prediction algorithms based on identified predictors.

**(I) Studies on VRE Related Risk Factors:** A number of studies assessed the risk factors associated VRE colonization at (or within two days) of hospital admission and developed risk-score based prediction rules **(Furuno et al. 2004)** **(Tacconelli et al. 2004)** **(Morgan et al. 2010)** **(Warren et al. 2003)**. The significant predictors identified by these studies were (i) prior hospital and/or prior ICU stay, (ii) antibiotics use within a year, (iii) chronic renal failure with dialysis or long-term hemodialysis use, (iv) LTCF stay, (v) age ≥ 60, and (vi) prior colonization with MRSA. Yoon et al. studied VRE colonization upon ICU admission and developed another prediction rule based on (1) ICU readmission during hospitalization, (2) chronic obstructive lung disease, and (3) recent vancomycin or another antibiotic use. Using a validation cohort of 1061 patients, they tested the performance of their and other four prediction methods, where their method achieved the highest AUC (0.90) and 88% sensitivity with 84% specificity **(Yoon et al. 2012)**.

**(II) Studies on MRSA Related Risk Factors:** Based on 16,950 ICU admission in 2010, collected from 147 ICUs in Spain, Callejo-Torre et. al **(Callejo-Torre et al. 2016)** performed a multivariable logistic regression analysis for MRSA colonization and infection upon ICU admission, and identified being male or trauma critical patient, receipt of urgent surgery, use of immunosuppression, being admitted from other ICUs, wards, or LTCF, and having a skin-soft tissue infection as predictors. The AUC-ROC was 0.77, sensitivity was 67%, and specificity was 76.5% in the testing set (2/3 of the whole data), and the AUC-ROC was 0.72 in the validation set (1/3 of the data). A recent observational and 1-year prospective longitudinal study identified APACHE-II score, accounting for twelve physiologic variables, age, and underlying health, > 15 and hospital stay (prior ICU admission) > 4 days as the main predictors for MRSA colonization upon ICU admission **(Ochotorena et al. 2019)**. Focusing on previously unknown asymptomatic cases, a 7-month, prospective case-controlled study found that MRSA colonization at admission is positively correlated with age > 75, recent (6-month) use of antibiotics, prior hospitalization (also identified by other studies **(Riedel et al. 2008) (Haley et al. 2007)**) or intravenous therapy, urinary catheter procedure at admission and intra-hospital transfer and developed a risk score algorithm based on these six variables **(Harbarth et al. 2006)**. Developing a risk score algorithm based on these six variables, the authors calculated that the screening volume would reduce > 30% if the patients with low score are excluded, and 86% sensitivity would be achieved over the remaining population via MRSA surveillance upon admission.

A meta-analysis of seventy-six studies, including a total of 24,230 patients, established a clear association between antibiotic exposure and MRSA colonization **(Tacconelli et al. 2007)**. Another risk score algorithm, based on the predictors nursing home residence, diabetes, past-year hospitalization, and chronic skin condition, achieved sensitivity = 71% in the derivation set and 54% in the validation set and reduced screening load by 80% when cut-off score of 8 or greater is applied, which corresponds to the presence of past-year hospitalization or nursing home residence and at least one other predictor **(Torres and Sampathkumar 2013**). Using multivariable logistic regression, Robicsek et al. developed five prediction models with degrees of simplicity (i.e., number of variables) in a population of 23,314 patients (not previously known to be colonized) and tested these methods, as well as two algorithms developed by other researchers **(Harbarth et al. 2008)** **(Furuno et al. 2006)**, in a validation cohort of 26,650 patients **(Robicsek et al. 2011)**. The sensitivity of their models were higher than other two algorithms and were in the range of 68% and 62% in the validation set, which were achieved by testing only 30% of the patients for MRSA colonization within 1 day of hospital admission.

**(III) Studies on CRE Related Risk Factors:** A meta-analysis **(van Loon, Voor, and Vos 2018)** based on 74 studies found that significant risk factors associated with CRE presence (colonization or infection) include *(i)* the use of medical devices (e.g., biliary, venous or urinary catheter **(Schechner et al. 2013; Papadimitriou-Olivgeris et al. 2013; Borer et al. 2012; European Centre for Disease Prevention and Control 2011)**, mechanical ventilation **(Patel G et al. 2008)**), *(ii)* antibiotic exposure (e.g., carbapenem **(Patel G et al. 2008; Hussein K. et al. 2009; Song J.Y. and I.S. 2018)**, cephalosporin **(Song J.Y. and I.S. 2018; Patel G et al. 2008)**, glycopeptide, quinolone, *β*-lactam), *(iii)* invasive surgical/medical procedures (e.g., organ or stem-cell transplantation **(Patel G et al. 2008)**), *(iv)* having an underlying disease or condition (e.g., diabetes mellitus **(Schechner et al. 2013; Borer et al. 2012)** and renal **(Kofteridis et al. 2014)** or liver disease **(Salomão et al. 2017)**), *(v)* hospital (and in particular, ICU) admission **(Schechner et al. 2013; Kofteridis et al. 2014; Giannella et al. 2014)**, and (iv) certain patient characteristics such as poor functional status **(Schwaber et al. 2008)** or high Charlson Comorbidity Index score **(Miller and Johnson 2016)**. Other predictors for CRE presence that were identified based on the systematic review and expert panel discussions conducted and hosted by the European Centre for Disease Prevention and Control (ECDC) are *(i)* increased length of stay in a hospital, *(ii)* patient transfer within the same hospital **(European Centre for Disease Prevention and Control 2011)**, *(iii)* recent ( < 12 months) receipt of chemotherapy or use of dialysis machine, *(iv)* previous CRE carriage within < 12 months, and *(v)* epidemiologically linked contact with a known CRE carrier patient (e.g., use of the same bed **(Papadimitriou-Olivgeris et al. 2013)**) **(Magiorakos et al. 2017)**.

Multivariable logistic regression models have been utilized by numerous studies to identify the risk factors associated with CRE acquisition (colonization or infection) **(van Loon, Voor, and Vos 2018)**. Few studies employed logistic regression not only to identify predictors but also make predictions. Sullivan et al. (2018) built a logistic regression model to rapidly predict carbapenem resistance in patients with *Klebsiella pneumoniae* bacteremia, and validated their model with K-fold cross-validation **(Sullivan et al. 2018)**. Converting the logistic regression coefficients of the identified predictors into risk scores, several studies developed risk score algorithms to predict and classify patients at high risk for CRE acquisition **(Martin et al. 2013; Miller and Johnson 2016)**. Two studies used Regional Healthcare Ecosystem Analyst (RHEA) simulation model **(Lee et al. 2013)** on Orange County, California healthcare facilities to estimate the financial burden of CRE and investigate the effectiveness of CDC’s CRE toolkit on stopping the spread of CRE **(Lee et al. 2016; Bartsch et al. 2017)**. Using the RHEA model, Lee et al. (2015) showed that CRE would become endemic within 10 years in Orange County facilities if no intervention measure is put in place, and the CDC’s CRE toolkit, if implemented, effectively limits the spread CRE (yet not completely eradicate it) **(Lee et al. 2016)**. Bartsch et al. (2018) also employed the RHEA model on Orange County, and demonstrated that the active screening for CRE colonization and infection, as recommended by the CDC’s CRE toolkit, leads to significant cost savings, which is amplified if hospitals collectively implemented the CDC’s toolkit in a coordinated manner **(Bartsch et al. 2017)**.

**Bibliography:**

Bartsch, S.M., J.A. McKinnell, L.E. Mueller, L.G. Miller, S.K. Gohil, S.S. Huang, and B.Y. Lee. 2017. 'Potential economic burden of carbapenem-resistant Enterobacteriaceae (CRE) in the United States', *Clinical Microbiology and Infection*, 23: 48-e9.

Borer, A., L. Saidel-Odes, S. Eskira, R. Nativ, K. Riesenberg, I. Livshiz-Riven, F. Schlaeffer, M. Sherf, and N. Peled. 2012. 'Risk factors for developing clinical infection with carbapenem-resistant Klebsiella pneumoniae in hospital patients initially only colonized with carbapenem-resistant K pneumoniae', *American journal of infection control*, 40: 421-25.

Callejo-Torre, F., J.M.E. Bouza, P.O. Astigarraga, M.J.C. Del Corral, M.P. Martínez, and F. Alvarez-Lerma. 2016. 'Risk factors for methicillin-resistant Staphylococcus aureus colonisation or infection in intensive care units and their reliability for predicting MRSA on ICU admission', *Europe*, 5: 6.

European Centre for Disease Prevention and Control. 2011. 'Risk assessment on the spread of carbapenemase-producing Enterobacteriaceae (CPE) through patient transfer between healthcare facilities, with special emphasis on cross-border transfer'.

Furuno, J.P., A.D. Harris, M.O. Wright, J.C. McGregor, R.A. Venezia, J. Zhu, and E.N. Perencevich. 2004. 'Prediction rules to identify patients with methicillin-resistant Staphylococcus aureus and vancomycin-resistant enterococci upon hospital admission', *American journal of infection control*, 32: 436-40.

Furuno, J.P., J.C. McGregor, A.D. Harris, J.A. Johnson, J.K. Johnson, P. Langenberg, R.A. Venezia, J. Finkelstein, D.L. Smith, S.M. Strauss, and E.N. Perencevich. 2006. 'Identifying groups at high risk for carriage of antibiotic-resistant bacteria', *Archives of internal medicine*, 166: 580-85.

Giannella, M., E.M. Trecarichi, F.G. De Rosa, V. Del Bono, M. Bassetti, R.E. Lewis, A.R. Losito, S. Corcione, C. Saffioti, M. Bartoletti, and G. Maiuro. 2014. 'Risk factors for carbapenem‐resistant K lebsiella pneumoniae bloodstream infection among rectal carriers: a prospective observational multicentre study', *Clinical Microbiology and Infection*, 20: 1357-62.

Haley, C.C., D. Mittal, A. LaViolette, S. Jannapureddy, N. Parvez, and R.W. Haley. 2007. 'Methicillin-resistant Staphylococcus aureus infection or colonization present at hospital admission: multivariable risk factor screening to increase efficiency of surveillance culturing', *Journal of clinical microbiology,*, 45: 3031-38.

Harbarth, S., H. Sax, C. Fankhauser-Rodriguez, J. Schrenzel, A. Agostinho, and D. Pittet. 2006. 'Evaluating the probability of previously unknown carriage of MRSA at hospital admission', *The American journal of medicine*, 119: 275-e15.

Harbarth, S., H. Sax, I. Uckay, C. Fankhauser, A. Agostinho, J.T. Christenson, G. Renzi, J. Schrenzel, and D. Pittet. 2008. 'A predictive model for identifying surgical patients at risk of methicillin-resistant Staphylococcus aureus carriage on admission', *Journal of the American College of Surgeons*, 207: 683-89.

Hussein K., Sprecher H., Mashiach T., Oren I., Kassis I., and Finkelstein R. 2009. 'Carbapenem resistance among Klebsiella pneumoniae isolates: Risk factors, molecular characteristics, and susceptibility patterns', *Infection Control & Hospital Epidemiology*, 30: 666-71.

Kofteridis, D.P., A. Valachis, D. Dimopoulou, S. Maraki, A. Christidou, E. Mantadakis, and G. Samonis. 2014. 'Risk factors for carbapenem-resistant Klebsiella pneumoniae infection/colonization: A case-case-control study', *Journal of Infection and Chemotherapy*, 20: 293-97.

Lee, B.Y., S.M. Bartsch, K.F. Wong, J.A. McKinnell, R.B. Slayton, L.G. Miller, C. Cao, D.S. Kim, A.J. Kallen, J.A. Jernigan, and S.S. Huang. 2016. 'The potential trajectory of carbapenem-resistant Enterobacteriaceae, an emerging threat to health-care facilities, and the impact of the Centers for Disease Control and Prevention toolkit', *American journal of epidemiology*, 183: pp.471-79.

Lee, B.Y., K.F. Wong, S.M. Bartsch, S.L. Yilmaz, T.R. Avery, S.T. Brown, Y. Song, A. Singh, D.S. Kim, and S.S. Huang. 2013. 'The Regional Healthcare Ecosystem Analyst (RHEA): a simulation modeling tool to assist infectious disease control in a health system', *Journal of the American Medical Informatics Association*, 20: e139-e46.

Magiorakos, A.P., K. Burns, J.R. Baño, M. Borg, G. Daikos, U. Dumpis, J.C. Lucet, M.L. Moro, E. Tacconelli, G.S. Simonsen, and E. Szilágyi. 2017. 'Infection prevention and control measures and tools for the prevention of entry of carbapenem-resistant Enterobacteriaceae into healthcare settings: guidance from the European Centre for Disease Prevention and Control', *Antimicrobial Resistance & Infection Control*, 6: 113.

Martin, E.T., R. Tansek, V. Collins, K. Hayakawa, O. Abreu-Lanfranco, T. Chopra, P.R. Lephart, J.M. Pogue, K.S. Kaye, and D. Marchaim. 2013. 'The carbapenem-resistant Enterobacteriaceae score: a bedside score to rule out infection with carbapenem-resistant Enterobacteriaceae among hospitalized patients', *American journal of infection control*, 41: 180-82.

Miller, B.M., and S.W. Johnson. 2016. 'Demographic and infection characteristics of patients with carbapenem-resistant Enterobacteriaceae in a community hospital: development of a bedside clinical score for risk assessment', *American journal of infection control*, 44: 134-37.

Morgan, D.J., H.R. Day, J.P. Furuno, A. Young, J.K. Johnson, D.D. Bradham, and E.N. Perencevich. 2010. 'Improving efficiency in active surveillance for methicillin-resistant Staphylococcus aureus or vancomycin-resistant Enterococcus at hospital admission', *Infection Control & Hospital Epidemiology*, 31: 1230-35.

Ochotorena, E., J.J. Hernández Morante, R. Cañavate, R.A. Villegas, and I. Viedma. 2019. 'Methicillin-resistant Staphylococcus aureus and other multidrug-resistant colonizations/infections in an intensive care unit: predictive factors', *Biological research for nursing*, 21: 190-97.

Papadimitriou-Olivgeris, M., M. Marangos, F. Fligou, M. Christofidou, C. Sklavou, S. Vamvakopoulou, E.D. Anastassiou, and K.S Filos. 2013. 'KPC-producing Klebsiella pneumoniae enteric colonization acquired during intensive care unit stay: the significance of risk factors for its development and its impact on mortality. ', *Diagnostic microbiology and infectious disease*, 77: 169-73.

Patel G, Huprikar S, Factor SH, Jenkins SG, and Calfee DP. 2008. 'Outcomes of carbapenem-resistant Klebsiella pneumoniae infection and the impact of antimicrobial and adjunctive therapies', *Infection Control & Hospital Epidemiology*, 29 1099 - 106.

Riedel, S., D. Von Stein, K. Richardson, J. Page, S. Miller, P. Winokur, and D. Diekema. 2008. 'Development of a prediction rule for methicillin-resistant Staphylococcus aureus and vancomycin-resistant enterococcus carriage in a Veterans Affairs Medical Center population', *Infection Control & Hospital Epidemiology*, 29: 969-71.

Robicsek, A., J.L. Beaumont, M.O. Wright, R.B. Thomson, K.L. Kaul, and L.R. Peterson. 2011. 'Electronic prediction rules for methicillin-resistant Staphylococcus aureus colonization', *Infection Control & Hospital Epidemiology*, 32: 9-19.

Salomão, M.C., T. Guimarães, D.F. Duailibi, M.B.M. Perondi, L.S.H. Letaif, A.C. Montal, F. Rossi, A.P. Cury, A.J.S. Duarte, A.S. Levin, and I. Boszczowski. 2017. 'Carbapenem-resistant Enterobacteriaceae in patients admitted to the emergency department: prevalence, risk factors, and acquisition rate', *Journal of Hospital Infection*, 97: 241-46.

Schechner, V., T. Kotlovsky, M. Kazma, H. Mishali, D. Schwartz, S. Navon-Venezia, M.J. Schwaber, and Y. Carmeli. 2013. ' Asymptomatic rectal carriage of blaKPC producing carbapenem-resistant Enterobacteriaceae: who is prone to become clinically infected?', *Clinical Microbiology and Infection*, 19: 451-56.

Schwaber, M.J., S. Klarfeld-Lidji, S. Navon-Venezia, D. Schwartz, A. Leavitt, and Y. Carmeli. 2008. 'Predictors of carbapenem-resistant Klebsiella pneumoniae acquisition among hospitalized adults and effect of acquisition on mortality', *Antimicrobial Agents and Chemotherapy*, 52: 1028-33.

Song J.Y., and Jeong I.S. 2018. 'Development of a risk prediction model of carbapenem-resistant Enterobacteriaceae colonization among patients in intensive care units', *American journal of infection control*.

Sullivan, T., O. Ichikawa, J. Dudley, L. Li, and J. Aberg. 2018. 'The rapid prediction of carbapenem resistance in patients with Klebsiella pneumoniae bacteremia using electronic medical record data', *In Open forum infectious diseases*, 5: ofy091. US: Oxford University Press.

Tacconelli, E., De Angelis, Cataldo G., Pozzi M.A., E., , and R. Cauda. 2007. 'Does antibiotic exposure increase the risk of methicillin-resistant Staphylococcus aureus (MRSA) isolation? A systematic review and meta-analysis', *Journal of antimicrobial chemotherapy*, 61: 26-38.

Tacconelli, E., A.W. Karchmer, D. Yokoe, and E.M. D'agata. 2004. 'Preventing the influx of vancomycin-resistant enterococci into health care institutions, by use of a simple validated prediction rule', *Clinical infectious diseases*, 39: 964-70.

Torres, K., and P. Sampathkumar. 2013. 'Predictors of methicillin-resistant Staphylococcus aureus colonization at hospital admission', *American journal of infection control*, 41: 1043-47.

van Loon, K., A.F. Voor, and M.C. Vos. 2018. 'A systematic review and meta-analyses of the clinical epidemiology of carbapenem-resistant Enterobacteriaceae', *Antimicrobial Agents and Chemotherapy*, 62: e01730-17.

Warren, D.K., M.H. Kollef, S.M. Seiler, S.K. Fridkin, and V.J. Fraser. 2003. 'The epidemiology of vancomycin-resistant Enterococcus colonization in a medical intensive care unit', *Infection Control & Hospital Epidemiology*, 24: 257-63.

Yoon, Y.K., H.J. Kim, W.J. Lee, S.E. Lee, K.S. Yang, D.W. Park, J.W. Sohn, and M.J. Kim. 2012. 'Clinical prediction rule for identifying patients with vancomycin-resistant enterococci (VRE) at the time of admission to the intensive care unit in a low VRE prevalence setting', *Journal of antimicrobial chemotherapy*, 67: 2963-69.

**Appendix C: Odds Ratio and Feature Importance Values**

**Appendix D: Sample Programming Codes for the Machine Learning Algorithms**

**D.1 FUNCTIONS, LIBRARIES, AND PACKAGES USED FOR PREDICTION**

*##############################################################################*
*################## IMPORT LIBRARIES AND PACKAGES ##################*

**import** **numpy** **as** **np**

**import** **pandas** **as** **pd**

**import** **copy**

**import** **matplotlib.pyplot** **as** **plt**

**import** **collections**

**import** **scipy**

**import** **seaborn** **as** **sns**

**import** **statsmodels.api** **as** **sm**

**import** **datetime**

**import** **time**

**from** **sklearn** **import** preprocessing

*##############################################################################*

*################## IMPORT LIBRARIES AND PACKAGES ##################*

%**pylab** inline

**import** **time**, **glob**, **pickle**

**import** **seaborn** **as** **sns**

**import** **pandas** **as** **pd**

**import** **sklearn** **as** **skl**

**import** **scipy.optimize** **as** **so**

**from** **sklearn.model_selection** **import** train_test_split, StratifiedKFold, GridSearchCV, cross_val_score, cross_val_predict

**from** **sklearn.linear_model** **import** LogisticRegressionCV, LogisticRegression

**from** **sklearn.tree** **import** DecisionTreeClassifier, export_graphviz

**from** **sklearn.ensemble** **import** RandomForestClassifier, GradientBoostingClassifier

**from** **sklearn.ensemble.partial_dependence** **import** partial_dependence, plot_partial_dependence

**from** **sklearn.metrics** **import** make_scorer, brier_score_loss, roc_auc_score

*##############################################################################*

*################## FUNCTION tp_rate ##################*

*# Create function to compute true positives for given threshold*

**def** tp_rate(p, t=0.5):

**return** sum(np.where(p > t, 1, 0)) / float(len(p))

*##############################################################################*

*################## FUNCTION sensitivity ##################*

*# Create function to compute sensitivity for given threshold*

**def** sensitivity(p, labels, t=0.5):

*# Map predicted probabilities to class labels*

pc = np.where(p >= t, 1, 0)

*# Calculate negative sensitivity*

**return** -float(sum(pc * labels)) / sum(labels)

*##############################################################################*

*################## FUNCTION sensitivity_opt ################*

*# Create function to compute sensitivity for given threshold*

**def** sensitivity_opt(t, labels, p):

*# Map predicted probabilities to class labels*

pc = np.where(p >= t, 1, 0)

*# Calculate negative sensitivity*

**return** -float(sum(pc * labels)) / sum(labels)

*##############################################################################*

*################## FUNCTION specificity ###################*

*# Create function to compute specificity for given threshold*

**def** specificity(p, labels, t=0.5):

*# Map predicted probabilities to class labels*

pc = np.where(p >= t, 1, 0)

*# Calculate negative specificity*

**return** -float(np.sum(np.logical_not(pc) * np.logical_not(labels))) / sum(np.logical_not(labels))

*##############################################################################*

*################## FUNCTION acc ###################*

*# Create function to compute accuracy for given threshold*

**def** acc(p, labels, t=0.5):

*# Map predicted probabilities to class labels*

pc = np.where(p >= t, 1, 0)

*# Calculate negative accuracy*

**return** -float(np.sum(pc == labels)) / len(labels)

*##############################################################################*

*################## FUNCTION youden ###################*

*# Create function to compute Youden's index for given threshold*

**def** youden(p, labels, t=0.5):

*# Calculate negative Youden's index*

**return** sensitivity(p, labels, t) + specificity(p, labels, t) + 1

*##############################################################################*

*################## FUNCTION youden_opt ###################*

*# Create function for optimizing Youden's index for given threshold*

**def** youden_opt(t, labels, p):

*# Calculate negative Youden's index*

**return** sensitivity(p, labels, t) + specificity(p, labels, t) + 1

*##############################################################################*

*################## FUNCTION precision ###################*

*# Create function to compute precision (positive predictive value)*

**def** precision(p, labels, t=0.5):

*# Map predicted probabilities to class labels*

pc = np.where(p >= t, 1, 0)

*# Calculate negative precision*

**return** -float(sum(pc * labels)) / sum(pc) **if** sum(pc) **else** 0

*##############################################################################*

*################## FUNCTION precision_opt ###################*

*# Create function for optimizing precision (positive predictive value)*

**def** precision_opt(t, labels, p):

*# Map predicted probabilities to class labels*

pc = np.where(p >= t, 1, 0)

*# Calculate negative precision*

**return** -float(sum(pc * labels)) / sum(pc) **if** sum(pc) **else** 0

*##############################################################################*

*################## FUNCTION f1score ###################*

*# Create function to compute F1-score for given threshold*

**def** f1score(t, p, labels):

*# Calculate negative f1-score*

recall = -sensitivity(t, p, labels)

prec = -precision(t, p, labels)

**return** -2 * prec * recall / (prec + recall)

*##############################################################################*

*################## FUNCTION thresh_opt ###################*

*# Define function for optimizing binary classification thresholds*

**def** thresh_opt(labels, p, fun):

opt = so.minimize_scalar(fun, method='bounded', bounds = (0,0.05),

args=(labels, p[:,1]), options={'maxiter': 2000})

**return** opt.x

*##############################################################################*

*################## FUNCTION summarize_perf ###################*

*# Define function to summarize performance results*

**def** summarize_perf(y, y_pred, thresh=0.5):

R = {'AUC': roc_auc_score(y, y_pred),

'Sensitivity': -sensitivity(y_pred, y, t=thresh),

'Specificity': -specificity(y_pred, y, t=thresh),

'Accuracy': -acc(y_pred, y, t=thresh),

'Precision': -precision(y_pred, y, t=thresh),

'Youdens Index': -youden(y_pred, y, t=thresh),

'TP Rate': tp_rate(y_pred, t=thresh)}

R['Brier Score'] = brier_score_loss(y, y_pred)

R = pd.DataFrame(R, index=[0])[['AUC', 'Sensitivity', 'Specificity', 'Brier Score', 'Accuracy', 'Precision','Youdens Index', 'TP Rate']]

**return** R

*##############################################################################*

*################## FUNCTION partial_dependence ###################*

*# Define function to calculate partial dependence*

**def** partial_dependence(model, X, preds, pred, values=**None**, cat=**False**):

*# Determine index/indices of predictor*

**if** cat:

*# Categorical variable, search preds for all categories*

bool = map(**lambda** s: pred **in** s, preds)

values, ip = zip(*[(re.search(re.escape(pred) + r"(.+)", p).group(1), k) **for** k, (p, b) **in** enumerate(zip(preds, bool)) **if** b])

**else**:

*# Numerical variable*

ip = preds.index(pred)

*# Loop through values of variable and average predictions*

ser = pd.Series()

**for** j, val **in** enumerate(values):

*# Update predictor array*

**if** isinstance(ip, int):

*# Update single value - Numerical predictor*

X[:, ip] = val

**else**:

*# Update multiple values - Categorical predictor*

**for** i **in** ip:

**if** val **in** preds[i]:

X[:, i] = 1

**else**:

X[:, i] = 0

*# Generate predictions*

ser = ser.set_value(val, model.predict_proba(X)[:,1].mean())

**return** ser

*##############################################################################*

*################## FUNCTION output ###################*

*# Define function to output pickled model objects*

**def** output(model, fname):

**with** open(fname + '_model.pickle', 'wb') **as** f:

pickle.dump(model, f)

print( "File Written" )

*##############################################################################*

*# Define threshold optimization score functions*

thresh_scorer_prec = make_scorer(thresh_opt, greater_is_better=**True**, needs_proba=**True**, fun=precision_opt )

thresh_scorer_sens = make_scorer(thresh_opt, greater_is_better=**True**, needs_proba=**True**, fun=sensitivity_opt )

thresh_scorer_youd = make_scorer(thresh_opt, greater_is_better=**True**, needs_proba=**True**, fun=youden_opt )

thresh_scorer_f1 = make_scorer(thresh_opt, greater_is_better=**True**, needs_proba=**True**, fun=f1score )

**D.2 LOGISTIC REGRESSION ALGORITHM FOR CRE PREDICTION**

*##############################################################################*

*################# TRAINING & TEST DATASETS #################*

**if** Response_Variable.shape[0] != Explanatory_Variables.shape[0] :

Difference_Inconsistency = Explanatory_Variables.shape[0] - Response_Variable.shape[0]

**for** x **in** range( 0 , int( Difference_Inconsistency ) ):

Delete_The_Last_Row = Explanatory_Variables.shape[0]-1

Explanatory_Variables.drop(Explanatory_Variables.index[Delete_The_Last_Row], inplace=**True**)

*# Create stratified training and test splits*

N_Folds_Cross_Validation = 10

Random_Number_Generation_Seed = 1234

Xo = Explanatory_Variables

y = Response_Variable

Xo_train, Xo_test, y_train, y_test = train_test_split( Xo , y , test_size=0.2, random_state= Random_Number_Generation_Seed , stratify=y )

cv = StratifiedKFold(n_splits= N_Folds_Cross_Validation )

<class 'pandas.core.frame.DataFrame'>

RangeIndex: 3661 entries, 0 to 3660

Columns: 413 entries, Admit_Source_0 to Total_Recent_Procedures_5

dtypes: uint8(413)

memory usage: 1.4 MB

In [2]:

*##############################################################################*

*####### K-Fold Cross Validation: Lasso Logistic Regression ########*

olasso = LogisticRegressionCV(Cs=100, cv=cv, penalty='l1', solver='liblinear', scoring='roc_auc')

%**time** olasso.fit(Xo_train, y_train)

CPU times: user 11min 53s, sys: 3.93 s, total: 11min 57s

Wall time: 12min 20s

Out[2]:

LogisticRegressionCV(Cs=100, class_weight=None,

cv=StratifiedKFold(n_splits=10, random_state=None, shuffle=False),

dual=False, fit_intercept=True, intercept_scaling=1.0,

max_iter=100, multi_class='ovr', n_jobs=1, penalty='l1',

random_state=None, refit=True, scoring='roc_auc',

solver='liblinear', tol=0.0001, verbose=0)

In [3]:

print("Best score:", olasso.scores_[1].mean(axis=0).max())

Best score: 0.707895731209

In [4]:

plt.figure(figsize=(6,4))

plt.semilogx(olasso.Cs_, olasso.scores_[1].mean(axis=0), 'k.')

plt.xlabel('C')

plt.ylabel('AUC')

Out[4]:


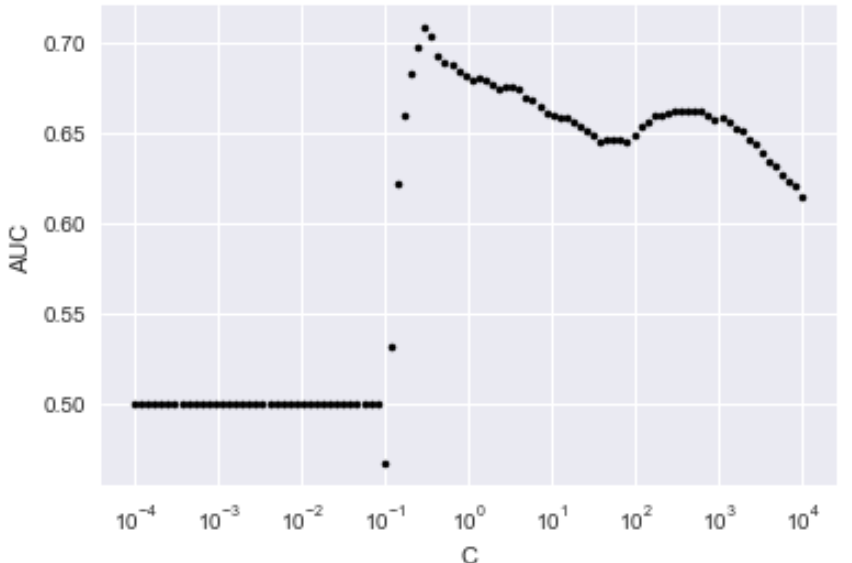


In [5]:

olr = LogisticRegression(penalty='l1', C = olasso.C_[0])

olr.fit(Xo_train, y_train)

*# Summarize predictor coefficients*

k = 1

cs_olr = pd.DataFrame({'Coefficients': olr.coef_[0]}, index=Categorical_Columns_Extended_CRE)

cs_olr.loc['Intercept'] = olr.intercept_

cs_olr['Odds Ratio'] = np.exp(cs_olr['Coefficients'])

cs_olr.to_csv('coefs_olr' + str(k) + '_new.csv', index=**True**)

cs_olr

Out[5]:

|  | **Coefficients** | **Odds Ratio** |
| --- | --- | --- |
| **Admit_Source_1** | -0.199691 | 0.818984 |
| **Admit_Source_2** | -0.259425 | 0.771495 |
| **Admit_Source_3** | 0.000000 | 1.000000 |
| **Admit_Source_4** | 0.000000 | 1.000000 |
| **Admit_Source_5** | 0.000000 | 1.000000 |
| **Admit_Source_6** | 0.000000 | 1.000000 |
| **Admit_Source_7** | 0.000000 | 1.000000 |
| **Admit_Source_8** | 0.000000 | 1.000000 |
| **Admit_Source_9** | 0.000000 | 1.000000 |
| **Admit_Source_10** | 0.000000 | 1.000000 |
| **Admit_Source_11** | 0.000000 | 1.000000 |
| **Admit_Source_12** | 0.000000 | 1.000000 |
| **Admit_Source_13** | 0.000000 | 1.000000 |
| **Admit_Source_14** | 0.000000 | 1.000000 |
| **Admit_Source_15** | 0.000000 | 1.000000 |
| **Admit_Source_16** | 0.000000 | 1.000000 |
| **Admit_Type_1** | 0.000000 | 1.000000 |
| **Admit_Type_2** | 0.000000 | 1.000000 |
| **Admit_Type_3** | 0.000000 | 1.000000 |
| **Admit_Type_4** | 0.000000 | 1.000000 |
| **Admit_Type_5** | 0.000000 | 1.000000 |
| **Admit_Type_6** | 0.000000 | 1.000000 |
| **Antibiotic_amino_1** | 0.000000 | 1.000000 |
| **...** | ... | ... |
| **Recent_ICD_Class_Double_5_1** | 0.128918 | 1.137597 |
| **Recent_ICD_Class_Double_6_1** | 0.000000 | 1.000000 |
| **Recent_ICD_Class_Double_7_1** | 0.000000 | 1.000000 |
| **Recent_ICD_Class_Double_8_1** | 0.000000 | 1.000000 |
| **Recent_ICD_Class_Double_9_1** | 0.000000 | 1.000000 |
| **Recent_ICD_Class_Single_0_1** | 0.000000 | 1.000000 |
| **Recent_ICD_Class_Single_1_1** | 0.000000 | 1.000000 |
| **Recent_ICD_Class_Single_2_1** | 0.000000 | 1.000000 |
| **Recent_ICD_Class_Single_3_1** | 0.176938 | 1.193557 |
| **Recent_ICD_Class_Single_4_1** | 0.000000 | 1.000000 |
| **Recent_ICD_Class_Single_5_1** | 0.592908 | 1.809242 |
| **Recent_ICD_Class_Single_6_1** | 0.000000 | 1.000000 |
| **Recent_ICD_Class_Single_7_1** | 0.000000 | 1.000000 |
| **Region_1** | 0.000000 | 1.000000 |
| **Region_2** | 0.000000 | 1.000000 |
| **Region_3** | 0.000000 | 1.000000 |
| **Total_Recent_Procedures_1** | -0.024017 | 0.976269 |
| **Total_Recent_Procedures_2** | 0.000000 | 1.000000 |
| **Total_Recent_Procedures_3** | 0.000000 | 1.000000 |
| **Total_Recent_Procedures_4** | 0.000000 | 1.000000 |
| **Total_Recent_Procedures_5** | 0.000000 | 1.000000 |
| **Intercept** | -4.234094 | 0.014493 |

235 rows × 2 columns

In [6]:

Logistic_Regression_CRE = pd.DataFrame( cs_olr )

Logistic_Regression_CRE.to_csv( "Logistic_Regression_CRE.csv" )

*# Optimize threshold*

threshes_olr = cross_val_score(olr, Xo_train, y_train, cv=cv, scoring=thresh_scorer_youd)

print( np.mean(threshes_olr), threshes_olr )

0.0206874683872 [ 0.01675324 0.01208848 0.01908629 0.02340371 0.03090585 0.01954743

0.0159576 0.02214377 0.01983873 0.02714959]

In [7]:

*# Generate out-of-sample predictions*

y_pred_olr = olr.predict_proba(Xo_test)[:,1]

sns.distplot(y_pred_olr)

plt.xlim([0,1])

plt.xlabel('Predicted Probabilities')

Out[7]:


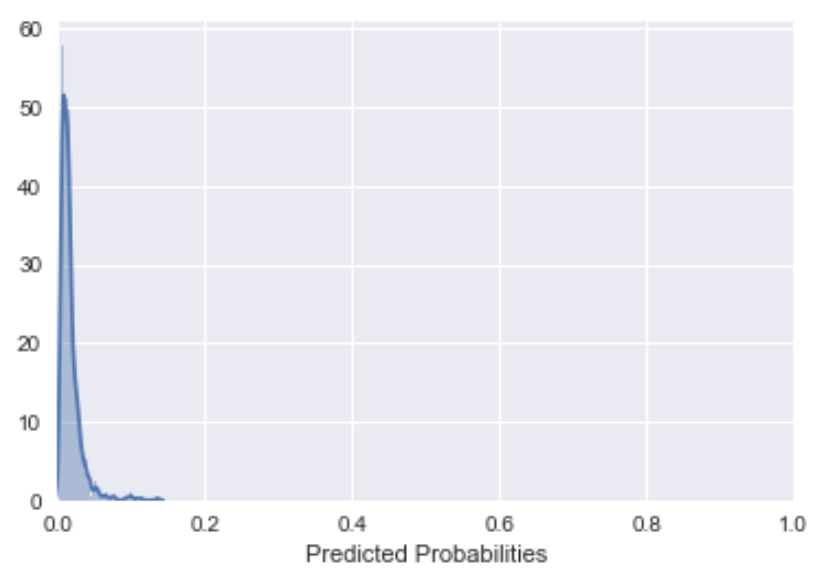


In [8]:

*# Summarize test performance of random forest model*

R_olr = summarize_perf(y_test, y_pred_olr, thresh=np.mean(threshes_olr))

R_olr

Out[8]:

|  | **AUC** | **Sensitivity** | **Specificity** | **Brier Score** | **Accuracy** | **Precision** | **Youdens Index** | **TP Rate** |
| --- | --- | --- | --- | --- | --- | --- | --- | --- |
| **0** | 0.757807 | 0.636364 | 0.778393 | 0.014711 | 0.776262 | 0.041916 | 0.414757 | 0.227831 |

In [9]:

*# Visualize predicted probability distribution with optimal threshold*

sns.set_style('ticks')

plt.figure(figsize=(6,2))

plt.plot(y_pred_olr, y_test, 'k.', markersize = 3, alpha=0.05)

plt.plot([0,1],[0,1], 'w.')

plt.yticks([0,1])

sns.despine(ax = plt.gca(), left=**False**, right=**True**, top=**False**, bottom = **False**, trim=**True**)

plt.gca().xaxis.set_ticks_position('bottom')

plt.hold(**True**)

plt.eventplot([np.mean(threshes_olr)], colors='k', lineoffsets=[0.5], linelengths=[2.0], linewidths=[0.5])

plt.xlim([0,1])

plt.ylim([-1,2])

plt.xlabel('Predicted Probability')

plt.ylabel('Class')

**D.3 RANDOM FOREST ALGORITHM FOR CRE PREDICTION**

*##############################################################################*

*################# TRAINING & TEST DATASETS #################*

**if** Response_Variable.shape[0] != Explanatory_Variables.shape[0] :

Difference_Inconsistency = Explanatory_Variables.shape[0] - Response_Variable.shape[0]

**for** x **in** range( 0 , int( Difference_Inconsistency ) ):

Delete_The_Last_Row = Explanatory_Variables.shape[0]-1

Explanatory_Variables.drop(Explanatory_Variables.index[Delete_The_Last_Row], inplace=**True**)

*# Create stratified training and test splits*

N_Folds_Cross_Validation = 10

Random_Number_Generation_Seed = 1234

Xo = Explanatory_Variables

y = Response_Variable

Xo_train, Xo_test, y_train, y_test = train_test_split( Xo , y , test_size=0.2, random_state= Random_Number_Generation_Seed , stratify=y )

cv = StratifiedKFold(n_splits= N_Folds_Cross_Validation )

Populating the interactive namespace from numpy and matplotlib

<class 'pandas.core.frame.DataFrame'>

RangeIndex: 3661 entries, 0 to 3660

Columns: 413 entries, Admit_Source_0 to Total_Recent_Procedures_5

dtypes: uint8(413)

memory usage: 1.4 MB

In [2]:

*##############################################################################*

*####### K-Fold Cross Validation: Random Forest ########*

*# Train random forest*

n_est = 200

param_grid={"min_samples_leaf": [ 5, 10 ,15 ,20 ,25 ,30 ,35 ,40 ,45 ,50 ,60 ,70 ,80 ,90 ,100 ,125 ,150 ,175 ,200 ,225 ,250 ] }

orf_grid = GridSearchCV(RandomForestClassifier(n_estimators=n_est, max_features='auto', criterion='gini'),

param_grid=param_grid, cv=cv, scoring='roc_auc', n_jobs=4)

%**time** orf_grid.fit(Xo_train, y_train)

CPU times: user 1.4 s, sys: 115 ms, total: 1.52 s

Wall time: 57.4 s

Out[2]:

GridSearchCV(cv=StratifiedKFold(n_splits=10, random_state=None, shuffle=False),

error_score='raise',

estimator=RandomForestClassifier(bootstrap=True, class_weight=None, criterion='gini',

max_depth=None, max_features='auto', max_leaf_nodes=None,

min_impurity_decrease=0.0, min_impurity_split=None,

min_samples_leaf=1, min_samples_split=2,

min_weight_fraction_leaf=0.0, n_estimators=200, n_jobs=1,

oob_score=False, random_state=None, verbose=0,

warm_start=False),

fit_params=None, iid=True, n_jobs=4,

param_grid={'min_samples_leaf': [5, 10, 15, 20, 25, 30, 35, 40, 45, 50, 60, 70, 80, 90, 100, 125, 150, 175, 200, 225, 250]},

pre_dispatch='2*n_jobs', refit=True, return_train_score='warn',

scoring='roc_auc', verbose=0)

In [3]:

*# Output grid search results*

print( orf_grid.best_params_, orf_grid.best_score_ )

{'min_samples_leaf': 20} 0.783197758638

In [4]:

*# Feature importance*

k=1

orf = orf_grid.best_estimator_

ofidf = pd.DataFrame({'Importance': orf.feature_importances_}, index=Categorical_Columns_Extended_CRE)

ofidf.to_csv('orf_featimpo' + str(k) + '_CRE.csv', index=**True**)

ofidf.sort_values(by='Importance', ascending=**False**)

Out[4]:

|  | **Importance** |
| --- | --- |
| **Recent_ICD_Class_Single_5_1** | 0.037290 |
| **Prior_ICU_4** | 0.036845 |
| **Recent_ICD_Class_Double_5_1** | 0.030831 |
| **LTCF_1** | 0.029178 |
| **Prior_Inpatient_5** | 0.021297 |
| **Recent_ICD_Class_Single_3_1** | 0.020631 |
| **Gender_1** | 0.019766 |
| **Recent_ICD_Class_Single_4_1** | 0.016484 |
| **Admit_Source_1** | 0.016400 |
| **Current_Total_Diagnosis_4** | 0.016309 |
| **Recent_ICD_Class_Double_1_1** | 0.016033 |
| **Current_Diagnosis_CCS_Class_2_1** | 0.015836 |
| **Antibiotic_fluoro_1** | 0.015414 |
| **Prior_CCS_Class_2_1** | 0.015148 |
| **Recent_ICD_Class_Double_7_1** | 0.015147 |
| **Prior_Antibiotic_1** | 0.015138 |
| **Total_Recent_Procedures_5** | 0.015014 |
| **Prior_ICD_Class_Double_4_1** | 0.014917 |
| **Race_1** | 0.014655 |
| **Prior_ICD_Class_Single_3_1** | 0.014537 |
| **Age_Group_4** | 0.014056 |
| **Recent_ICD_Class_Double_0_1** | 0.013883 |
| **Prior_CCS_Class_10_1** | 0.013631 |
| **Recent_ICD_Class_Double_3_1** | 0.013490 |
| **Total_Recent_Procedures_4** | 0.013268 |
| **Prior_ICD_Class_Double_7_1** | 0.012926 |
| **Current_Diagnosis_CCS_Class_5_1** | 0.012421 |
| **Prior_ICD_Class_Double_30_1** | 0.012158 |
| **Current_Diagnosis_CCS_Class_6_1** | 0.011888 |
| **Antibiotic_pen_1** | 0.011799 |
| **...** | ... |
| **Prior_ICD_Class_Double_37_1** | 0.000000 |
| **Prior_ICD_Class_Double_36_1** | 0.000000 |
| **Prior_ICD_Class_Double_35_1** | 0.000000 |
| **Prior_ICD_Class_Double_34_1** | 0.000000 |
| **Admit_Source_10** | 0.000000 |
| **Admit_Source_9** | 0.000000 |
| **Prior_CPT_Class_Detailed_10_1** | 0.000000 |
| **Prior_CPT_Class_Detailed_7_1** | 0.000000 |
| **Recent_ICD_Class_Double_22_1** | 0.000000 |
| **Prior_CPT_Class_Detailed_13_1** | 0.000000 |
| **Recent_ICD_Class_Double_19_1** | 0.000000 |
| **Prior_ICD_Class_Double_33_1** | 0.000000 |
| **Recent_ICD_Class_Double_15_1** | 0.000000 |
| **Prior_CPT_Class_Detailed_14_1** | 0.000000 |
| **Prior_CPT_Class_Detailed_16_1** | 0.000000 |
| **Prior_CPT_Class_Detailed_17_1** | 0.000000 |
| **Admit_Source_8** | 0.000000 |
| **Prior_CPT_Class_Detailed_18_1** | 0.000000 |
| **Prior_CPT_Class_Detailed_19_1** | 0.000000 |
| **Race_3** | 0.000000 |
| **Prior_CPT_Class_Detailed_2_1** | 0.000000 |
| **Prior_CPT_Class_Detailed_20_1** | 0.000000 |
| **Prior_CPT_Class_Detailed_21_1** | 0.000000 |
| **Prior_CPT_Class_Detailed_22_1** | 0.000000 |
| **Prior_CPT_Class_Detailed_3_1** | 0.000000 |
| **Prior_Multiple_Antibiotic_6** | 0.000000 |

234 rows × 1 columns

In [5]:

Random_Forest_CRE = pd.DataFrame( ofidf )

Random_Forest_CRE.to_csv( "Random_Forest_CRE.csv" )

In [6]:

*# Optimize threshold*

threshes_orf = cross_val_score(orf, Xo_train, y_train, cv=cv, scoring=thresh_scorer_youd)

print( np.mean(threshes_orf), threshes_orf )

0.0242167211133 [ 0.0192378 0.02243748 0.03818462 0.01575645 0.01083812 0.03833679

0.02482312 0.01480974 0.01180887 0.04593422]

In [7]:

*# Generate out-of-sample predictions*

y_pred_orf = orf.predict_proba(Xo_test)[:,1]

sns.distplot(y_pred_orf)

plt.xlim([0,1])

plt.xlabel('Predicted Probabilities')

Out[7]:


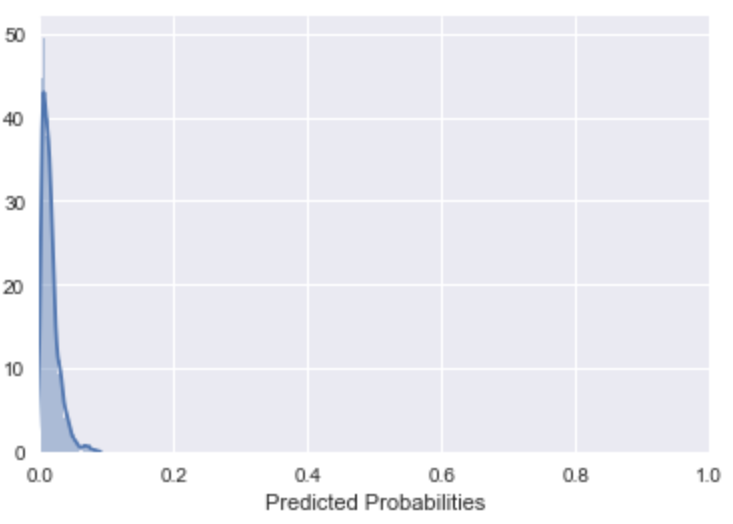


In [8]:

*# Summarize performance of random forest model*

R_orf = summarize_perf(y_test, y_pred_orf, thresh=np.mean(threshes_orf))

R_orf

Out[8]:

|  | **AUC** | **Sensitivity** | **Specificity** | **Brier Score** | **Accuracy** | **Precision** | **Youdens Index** | **TP Rate** |
| --- | --- | --- | --- | --- | --- | --- | --- | --- |
| **0** | 0.771972 | 0.545455 | 0.8241 | 0.014552 | 0.819918 | 0.045113 | 0.369554 | 0.181446 |

In [9]:

*# Visualize predicted probability distribution with optimal threshold*

sns.set_style('ticks')

plt.figure(figsize=(6,2))

plt.plot(y_pred_orf, y_test, 'k.', markersize = 3, alpha=0.05)

plt.plot([0,1],[0,1], 'w.')

plt.yticks([0,1])

sns.despine(ax = plt.gca(), left=**False**, right=**True**, top=**False**, bottom = **False**, trim=**True**)

plt.gca().xaxis.set_ticks_position('bottom')

plt.hold(**True**)

plt.eventplot([np.mean(threshes_orf)], colors='k', lineoffsets=[0.5], linelengths=[2.0], linewidths=[0.5])

plt.xlim([0,1])

plt.ylim([-1,2])

plt.xlabel('Predicted Probability')

plt.ylabel('Class')

In [ ]:

**D.4 XGBOOST ALGORITHM FOR CRE PREDICTION**

*##############################################################################*

*################# TRAINING & TEST DATASETS #################*

**if** Response_Variable.shape[0] != Explanatory_Variables.shape[0] :

Difference_Inconsistency = Explanatory_Variables.shape[0] - Response_Variable.shape[0]

**for** x **in** range( 0 , int( Difference_Inconsistency ) ):

Delete_The_Last_Row = Explanatory_Variables.shape[0]-1

Explanatory_Variables.drop(Explanatory_Variables.index[Delete_The_Last_Row], inplace=**True**)

*# Create stratified training and test splits*

N_Folds_Cross_Validation = 10

Random_Number_Generation_Seed = 1234

Xo = Explanatory_Variables

y = Response_Variable

Xo_train, Xo_test, y_train, y_test = train_test_split( Xo , y , test_size=0.2, random_state= Random_Number_Generation_Seed , stratify=y )

cv = StratifiedKFold(n_splits= N_Folds_Cross_Validation )

Populating the interactive namespace from numpy and matplotlib

<class 'pandas.core.frame.DataFrame'>

RangeIndex: 3661 entries, 0 to 3660

Columns: 413 entries, Admit_Source_0 to Total_Recent_Procedures_5

dtypes: uint8(413)

memory usage: 1.4 MB

In [2]:

*##############################################################################*

*####### K-Fold Cross Validation: XGBoost ########*

**import** **xgboost**

**from** **xgboost** **import** XGBClassifier

*##############*

*# Prevent libiomp5.dylib error when xgboost and matplotlib are both used simultaneously*

os.environ['KMP_DUPLICATE_LIB_OK']='True'

*##############*

*# Train XGBoost - Initialize Parameters*

n_est = 200

learn_rate = 0.05

depth_max = 5

min_weight_child = 1

gamma_value = 0.0

sub_sample = 0.8

column_sample_ratio = 0.8

objective_function = 'binary:logistic'

number_thread = 4

random_seed = 1337

*##############*

parameters = {'nthread' : [number_thread] ,

'objective' : ['binary:logistic'] ,

'learning_rate' : [learn_rate] ,

'max_depth' : [depth_max] ,

'min_child_weight': [min_weight_child] ,

'gamma' : [gamma_value] ,

'subsample' : [sub_sample] ,

'colsample_bytree': [column_sample_ratio] ,

'n_estimators' : [n_est] ,

'seed' : [random_seed] }

oxgboost_grid = GridSearchCV( XGBClassifier() , parameters , cv=cv , scoring='roc_auc', n_jobs=4 )

%**time** oxgboost_grid.fit(Xo_train, y_train)

CPU times: user 8.93 s, sys: 119 ms, total: 9.05 s

Wall time: 25.8 s

Out[2]:

GridSearchCV(cv=StratifiedKFold(n_splits=10, random_state=None, shuffle=False),

error_score='raise',

estimator=XGBClassifier(base_score=0.5, booster='gbtree', colsample_bylevel=1,

colsample_bytree=1, gamma=0, learning_rate=0.1, max_delta_step=0,

max_depth=3, min_child_weight=1, missing=None, n_estimators=100,

n_jobs=1, nthread=None, objective='binary:logistic', random_state=0,

reg_alpha=0, reg_lambda=1, scale_pos_weight=1, seed=None,

silent=True, subsample=1),

fit_params=None, iid=True, n_jobs=4,

param_grid={'nthread': [4], 'objective': ['binary:logistic'], 'learning_rate': [0.05], 'max_depth': [5], 'min_child_weight': [1], 'gamma': [0.0], 'subsample': [0.8], 'colsample_bytree': [0.8], 'n_estimators': [200], 'seed': [1337]},

pre_dispatch='2*n_jobs', refit=True, return_train_score='warn',

scoring='roc_auc', verbose=0)

In [3]:

*# Output grid search results*

print ( oxgboost_grid.best_params_, oxgboost_grid.best_score_ )

{'colsample_bytree': 0.8, 'gamma': 0.0, 'learning_rate': 0.05, 'max_depth': 5, 'min_child_weight': 1, 'n_estimators': 200, 'nthread': 4, 'objective': 'binary:logistic', 'seed': 1337, 'subsample': 0.8} 0.75205734025

In [4]:

*# Assign the tuned model*

oxgboost = oxgboost_grid.best_estimator_

oxgboost

*# Feature importance*

ofixgboost = pd.DataFrame({'Importance': oxgboost.feature_importances_}, index=Categorical_Columns_Extended_CRE)

ofixgboost.sort_values(by='Importance', ascending=**False**)

Out[4]:

|  | **Importance** |
| --- | --- |
| **Recent_ICD_Class_Double_5_1** | 0.031125 |
| **Recent_ICD_Class_Single_5_1** | 0.027568 |
| **Prior_CCS_Class_10_1** | 0.027123 |
| **Gender_1** | 0.024455 |
| **Prior_CCS_Class_2_1** | 0.022677 |
| **LTCF_1** | 0.020898 |
| **Current_Diagnosis_CCS_Class_5_1** | 0.020454 |
| **Current_Diagnosis_CCS_Class_2_1** | 0.018675 |
| **Current_Total_Diagnosis_4** | 0.018230 |
| **Nurse_1** | 0.018230 |
| **Prior_ICU_4** | 0.018230 |
| **Current_Diagnosis_CCS_Class_6_1** | 0.017786 |
| **Prior_ICU_1** | 0.017341 |
| **Recent_ICD_Class_Double_8_1** | 0.016896 |
| **Current_Total_Diagnosis_3** | 0.016896 |
| **Recent_ICD_Class_Single_4_1** | 0.016007 |
| **Recent_ICD_Class_Single_3_1** | 0.016007 |
| **Race_1** | 0.016007 |
| **Antibiotic_pen_1** | 0.015562 |
| **Age_Group_3** | 0.015562 |
| **Recent_ICD_Class_Double_7_1** | 0.015118 |
| **Prior_CCS_Class_8_1** | 0.015118 |
| **Age_Group_4** | 0.015118 |
| **Prior_Multiple_Antibiotic_3** | 0.014229 |
| **Recent_ICD_Class_Double_2_1** | 0.014229 |
| **Total_Recent_Procedures_3** | 0.014229 |
| **Total_Recent_Procedures_4** | 0.013339 |
| **Admit_Type_1** | 0.012450 |
| **Recent_ICD_Class_Double_3_1** | 0.012005 |
| **Prior_ICD_Class_Double_4_1** | 0.012005 |
| **...** | ... |
| **Prior_ICD_Class_Double_20_1** | 0.000000 |
| **Prior_ICD_Class_Double_16_1** | 0.000000 |
| **Prior_ICD_Class_Double_19_1** | 0.000000 |
| **Admit_Type_5** | 0.000000 |
| **Prior_CPT_Class_Detailed_5_1** | 0.000000 |
| **Admit_Source_13** | 0.000000 |
| **Prior_Total_Procedures_5** | 0.000000 |
| **Recent_ICD_Class_Double_18_1** | 0.000000 |
| **Recent_ICD_Class_Double_15_1** | 0.000000 |
| **Recent_ICD_Class_Double_14_1** | 0.000000 |
| **Prior_CPT_Class_Detailed_17_1** | 0.000000 |
| **Prior_CPT_Class_Detailed_18_1** | 0.000000 |
| **Admit_Source_14** | 0.000000 |
| **Prior_ICD_Class_Double_18_1** | 0.000000 |
| **Race_3** | 0.000000 |
| **Race_2** | 0.000000 |
| **Admit_Source_15** | 0.000000 |
| **Prior_CPT_Class_Detailed_2_1** | 0.000000 |
| **Prior_ICD_Class_Single_6_1** | 0.000000 |
| **Prior_Multiple_Antibiotic_6** | 0.000000 |
| **Prior_CPT_Class_Detailed_20_1** | 0.000000 |
| **Admit_Source_16** | 0.000000 |
| **Prior_CPT_Class_Detailed_21_1** | 0.000000 |
| **Prior_CPT_Class_Detailed_22_1** | 0.000000 |
| **Prior_Inpatient_2** | 0.000000 |
| **Prior_Inpatient_1** | 0.000000 |
| **Prior_ICU_3** | 0.000000 |
| **Prior_CPT_Class_Detailed_3_1** | 0.000000 |
| **Admit_Type_4** | 0.000000 |
| **Current_Diagnosis_CCS_Class_3_1** | 0.000000 |

234 rows × 1 columns

In [5]:

XGBoost_CRE = pd.DataFrame( ofixgboost )

XGBoost_CRE.to_csv( "XGBoost_CRE.csv" )

*# Optimize threshold*

threshes_oxgboost = cross_val_score( oxgboost , Xo_train , y_train , cv=cv , scoring = thresh_scorer_youd )

print( np.mean(threshes_oxgboost), threshes_oxgboost )

0.0253278025716 [ 0.0061126 0.03820027 0.01389649 0.01848505 0.04511634 0.04139252

0.01910287 0.02014362 0.00811951 0.04270877]

In [6]:

*# Generate out-of-sample predictions*

y_pred_oxgboost = oxgboost.predict_proba(Xo_test)[:,1]

sns.distplot(y_pred_oxgboost)

plt.xlim([0,1])

plt.xlabel('Predicted Probabilities')

Out[6]:


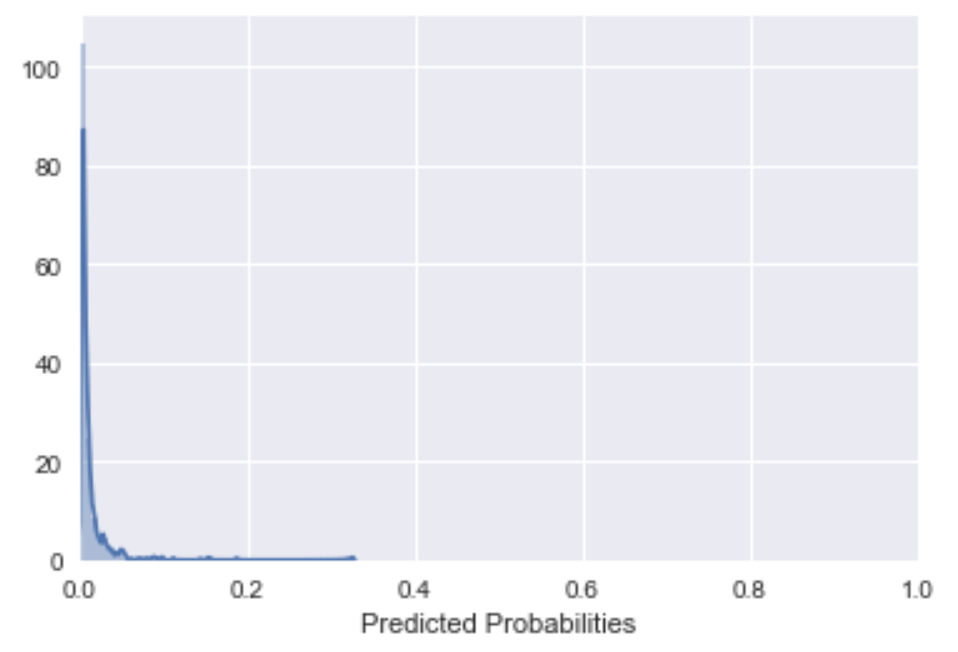


In [7]:

*# Summarize performance of XGBoost model*

R_oxgboost = summarize_perf( y_test, y_pred_oxgboost, thresh=np.mean(threshes_oxgboost) )

R_oxgboost

Out[7]:

|  | **AUC** | **Sensitivity** | **Specificity** | **Brier Score** | **Accuracy** | **Precision** | **Youdens Index** | **TP Rate** |
| --- | --- | --- | --- | --- | --- | --- | --- | --- |
| **0** | 0.757114 | 0.363636 | 0.915512 | 0.014747 | 0.907231 | 0.061538 | 0.279149 | 0.088677 |

In [8]:

*# Visualize predicted probability distribution with optimal threshold*

sns.set_style('ticks')

plt.figure(figsize=(6,2))

plt.plot(y_pred_oxgboost, y_test, 'k.', markersize = 3, alpha=0.05)

plt.plot([0,1],[0,1], 'w.')

plt.yticks([0,1])

sns.despine(ax = plt.gca(), left=**False**, right=**True**, top=**False**, bottom = **False**, trim=**True**)

plt.gca().xaxis.set_ticks_position('bottom')

plt.hold(**True**)

plt.eventplot([np.mean(threshes_oxgboost)], colors='k', lineoffsets=[0.5], linelengths=[2.0], linewidths=[0.5])

plt.xlim([0,1])

plt.ylim([-1,2])

plt.xlabel('Predicted Probability')

plt.ylabel('Class')
